# Combining ability and gene action for yield improvement in kenaf (*Hibiscus cannabinus* L.) under tropical conditions through diallel mating design

**DOI:** 10.1038/s41598-022-13529-y

**Published:** 2022-06-10

**Authors:** Md Al-Mamun, Mohd Y. Rafii, Azizah Binti Misran, Zulkarami Berahim, Zaiton Ahmad, Md Mahmudul Hasan Khan, Yusuff Oladosu

**Affiliations:** 1 Laboratory of Climate-Smart Food Crop Production, Institute of Tropical Agriculture and Food Security (ITAFoS), Universiti Putra Malaysia (UPM), 43400 UPM Serdang, Selangor Malaysia; 2 Bangladesh Jute Research Institute (BJRI), Dhaka, 1207 Bangladesh; 3 Department of Crop Science, Faculty of Agriculture, Universiti Putra Malaysia (UPM), 43400 UPM Serdang, Selangor Malaysia; 4 Agrotechnology and Bioscience Division, Malaysian Nuclear Agency, Bangi, 43000 Kajang, Selangor Malaysia; 5 Bangladesh Agricultural Research Institute (BARI), Gazipur, 1701 Bangladesh

**Keywords:** Developmental biology, Genetics, Plant sciences

## Abstract

Nine morphologically distinct kenaf genotypes were hybridized to produce 36 hybrids following a half diallel mating design. The combining ability and gene action of 15 yield and yield components were assessed in hybrids and their parents across two environments. Except for the mid diameter and plant height traits, there were highly significant differences (p ≤ 0.01) between the environments and the interaction of genotype and environment. Additive gene effects were considerable for the inheritance of these traits, and the expression of these additive genes was heavily influenced by the environment. Significant differences were found for all studied traits for GCA except top diameter and SCA except plant height and top diameter, implying the presence of both additive and non-additive gene action for the inheritance of the concerned characters. For all features except top diameter and number of nodes, the magnitude of GCA variation was significantly higher than that of SCA variance, indicating the additive gene's predominance. The parental lines P_1_, P_3_ and P_4_ were outstanding general combiners for fiber yield and yield-related parameters. Considering combining ability and genetic analysis study, the crosses P_1_ × P_4_, P_1_ × P_9_, P_2_ × P_3_, P_2_ × P_5_, P_4_ × P_6_, P_4_ × P_7_, P_4_ × P_9_, P_5_ × P_8_, and P_7_ × P_9_ were found promising for their heterotic response to higher fiber yield, stick yield, seed yield and could be for future improvement in kenaf breeding programmes.

## Introduction

Fiber crops have been a part of human society from the beginning of time. Humans have been known to gather raw materials from the wild for use as ropes or textiles throughout history. Societies later learnt how to cultivate these types of crops. Natural fibre crops are among the earliest known cultivated plants, and humans have continued to domesticate and improve upon them throughout human history. Fiber crop types have been extensively developed through effective selection and breeding in response to the requirements and values of various communities around the world^50^. Kenaf (*Hibiscus cannabinus* L., Malvaceae) is a high-value fibre crop with significant economic value (Keshk et al. 2006). It is widely cultivated for food and fibre, and it has been extensively utilized as a cordage crop to produce twine, rope, sackcloth, coarse, burlap, and fiberboard^50^. Kenaf is grown commercially for a variety of purposes, including pulping and paper production, oil spill bioremediation, livestock feed, and the production of biodegradable packaging materials^25^. Kenaf seed is also commercially important because it is a good oil source (16–22%) lubrication, soap production, cosmetics, linoleum, paints, and varnishes^8,33^.

In China, and India, hybrid kenaf cultivars accounted for most cultivated accessions^24^. Hybrid kenaf has received much attention due to its enhanced fiber quality and resistance to force^2^. Due to the ability of kenaf flowers to both self-pollinate and cross-pollinate, the crossover can range from 2 to 24%, depending on insect activity^4^. Dempsey^11^ reported that the productivity of F_1_ in kenaf ranges from 14 to 43% more than the parents. Compared to alternative mating designs, the diallel analysis is an effective method for screening parents for hybrid production. Different varieties of diallel crosses exist, however, in terms of the number of reciprocal crossings, half diallel crosses are more manageable for breeders than full diallel analysis^7^. Combining ability analysis is an effective tool for identifying superior hybrid parents with high general combining ability (GCA) and progenies with improved specific combining ability (SCA)^45^. It is also useful for measuring the genetic worth of parents and crossings in terms of gene activity in quantitative character inheritance and exploitation and breeding^13^.

General combining ability (GCA) refers to a parent's average performance in a series of crossings. In contrast, SCA refers to a hybrid combination that is better or worse than expected based on the average performance of the parental inbred lines involved^6^. Parents with a high GCA effect have additive gene activity, but they do not always have a favorable SCA in their combination^38^. Meanwhile, determining the sort of gene action that affects the phenotypes of interest using SCA estimation is useful in genetic research. A high SCA identifies non-additive gene action^46^. When measured in terms of average effects (components), SCA effects were bigger than GCA effects, indicating the importance of non-additive gene activity in influencing yield component expression^32^. SCA and GCA data aid in selecting hybrids and parents for successful breeding^34^. Therefore, strong hybrids are produced by parents who have good GCA^41^.

Heterosis is a genetic phenomenon caused by heterozygosity and is an important plant improvement measurement. Heterosis for fiber yield is well-known, and kenaf hybrid cultivars have been generated and used commercially in China^25^. The accumulation of dominant genes from both parents, according to Bruce^5^, is linked to the development of dominance heterosis. Yang et al.^48^ described epistasis as interactions among non-alleles on a genome, whereas Cordell^9^ defined it as interactions between genes. Jianmin et al.^20^ claimed that F_1_ heterosis can survive 1.4–1.7 generations on average, but that favorable hybrids could last 3–4 generations. By selecting genotypes with distinct genetic backgrounds, it will be highly useful for improving the variety of kenaf in the Malaysian tropical environment^12^. The primary goal of this research was to identify genotypes (parents and offspring) with good combining ability that could assist future kenaf improvement with high fiber yield.

## Materials and methods

### Planting materials

Nine kenaf genotype parents and 36 F_1_ hybrids were employed in this study (Table 1). Among the nine genotypes, one was a commercial variety from Bangladesh and eight mutant lines were developed from V-36 through acute and chronic gamma irradiation by the Malaysian Nuclear Agency in Bangi, Selangor. The parents were mated in all possible combinations, barring reciprocals (half diallel) at Field 10 in University Putra Malaysia to produce 36 F_1_ hybrids. The International Union for Conservation of Nature's (IUNC) statement on research involving species at risk of extinction and the convention on the trade in endangered species of wild flora and fauna were followed during the collection of plant materials and execution of the experiment. Table 1 contains a list of all of the accession numbers, along with their IDs codes.

**Table 1:** Origin and salient features of nine selected kenaf genotypes used as parents for diallel cross.

Parent	Accession	Mode of development	Generation	Salient features
P_1_	ML5	Acute (300), MNA	M_7_	Late maturing cultivar with high fiber yielding green palmate leaves and completely cream flower color with white stigma
P_2_	ML9	Acute (300), MNA	M_7_	Positive flowering attributes and deep green cordate leaves
P_3_	ML36-10	Acute (300), MNA	M_6_	Cordate leaves are pale green in color and produce a lot of fiber and stick
P_4_	ML36-24	Acute (1300), MNA	M_6_	Growing quickly and creating a lot of biomasses with high fiber and stick yield
P_5_	ML36-25	Acute (1300), MNA	M_6_	Best performer for both seed yield traits (seeds number per pod and 1000 seeds weight)
P_6_	ML36-27	Acute (1300), MNA	M_6_	Green stem with reddish patches and deep green cordate leaf
P_7_	BJRI Kenaf4	Conventional method, BJRI	Check	Purple stem with palmate leaf that develops swiftly and produces several pods per plant
P_8_	MLRing4P2	Chronic, MNA	M_6_	Bark thickness is comparatively low and the fiber to stick ratio is intermediate
P_9_	ML36-21(2)	Acute (800), MNA	M_6_	Green palmate leaves and are far less photosensitive

*MNA* Malaysian Nuclear Agency, *BJRI* Bangladesh Jute Research Institute.

### Hybridization techniques and raising of F_1_ seeds

Nine genotypes were chosen as parental materials and mated in diallel fashion omitting reciprocals, considering group distances, genetic distances, and other agronomic performance. To achieve flowering synchronization was observed during the crossing period where pure and healthy seeds were sown in the experiment field three times at 10-day intervals. The F_1_ seeds were collected after the fruits had ripened, using standard methods for emasculation and pollination.

### Experimental location

The experiment plots were conducted in two seasons in a humid tropical climate between latitude 2° 59′ N and longitude 101° 42′ E, at 48 m above sea level at Field 10, Universiti Putra Malaysia (UPM), Serdang, Selangor Darul Ehsan. The first season was conducted from June 2020 to September 2020, while the second was from March 2021 to June 2021.

### Experimental design and field layout

The field was mechanically ploughed and laddered for cultivation. Kenaf seeds from 45 entries, consisting of nine parents and 36 F_1_s were planted in peat moss soil in germination trays at a glasshouse for two weeks before being transplanted into the field plots of 59 m × 9 m. The experiment was laid out in Randomized Complete Block Design (RCBD) with three replications using a table of random numbers^15^. NPK Green (15:15:15) and NPK Blue (12:12:17) were applied at the prescribed dose of 450 kg per hectare shortly after seeding and after 40 days of transplanting. Intercultural operations like weeding, thinning, supplemental irrigations, and plant protection measures were performed appropriately throughout the cropping season.

### Data collection

The genetic diversity was assessed using 22 features (seven qualitative and 15 quantitative) related to the plant, flower, fiber yield, pod, and seed across two seasons. The observation was recorded from 10 randomly selected plants for each genotype per replication for each trait. The qualitative traits, including stem color and leaf shape were visually recorded at seedling and growth stages in the field. Leaf color, petiole color, pod shape, seed shape and seed coat color were visually recorded at the pre-bud and mature stages. Quantitative data collected include plant height, base diameter, core diameter, middle diameter, top diameter, nodes number, days to first flowering, days to 50% flowering, fresh stem weight with leaves and pods, fresh stem weight without leaves and pods, dry stick weight, dry fiber weight, pods number per plant, seeds number per pod and 1000 seeds weight (Table 2). Days to first flowering, days to 50% flowering, and pods number per plant were all verified in the field, and residual traits were measured in the lab 90 days after transplanting^3^.

**Table 2:** Quantitative characters studied from nine parents and their crosses.

Quantitative trait	Abbreviation (unit)	Description of evaluation method
Plant height	PH (cm)	Plant height was measured from the base to the tip of the main shoot of 10 randomly selected plants in meter scale at the time of harvest (pre-bud stage, 90 DAS)
Base diameter	BD (mm)	Average base diameter of 10 randomly selected plants was measured at the base of the stem in mm using slide calipers
Core diameter	CD (mm)	It was measured at the base point of the stem using slide calipers after the fiber separated from the stem. It was taken at the end of retting period
Middle diameter	MD (mm)	It was measured at the mid-point between base and top of the stem using slide calipers. It was also taken at the time of harvest
Top diameter	TD (mm)	It was measured at the top of the stem using slide calipers. It was also taken at the time of harvest
Number of nodes	NN	Number of nodes of 10 randomly selected plants was counted and averaged then for getting node no/plant
Days to first flowering	DTFF (days)	It was recorded as the number of days from the date of sowing to beginning of flowering in the population of each genotype
Days to 50% flowering	D50%F (days)	The number of days to flowering was quantified from planting date to the day when 50% flowering in the population of each genotype
Fresh stem weight with leaves and pods	FW1 (g)	Average fresh stem weight with leaves and pods of 10 plants was recorded using electric balance just after harvest and then per plant basis weight was calculated
Fresh stem weight without leaves and pods	FW2 (g)	Fresh stem weight of 10 randomly selected plants without leaves and pods was taken using electric balance just after harvest and then per plant basis weight was calculated
Dry stick weight	DSW (g)	It was measured from 10 previously selected plants after extraction of fiber and proper sun drying of the stick and the mean was computed
Dry fiber weight	DFW (g)	Dry fiber weight of randomly selected 10 plants from each plot was taken after retting and proper drying in the sun. Finally, per plant basis weight was calculated
Number of pods per plant	NF	Number of total pods from first to last pod setting per plant was counted in the field and recorded
Number of seeds per pod	NS	Number of matured seeds from 30 randomly selected pods per replication was counted and it was then averaged and replication wise recorded
1000 seeds weight	SW (g)	Weight of 1000 dry seeds (10% moisture) of each genotype for each replication was measured using digital balance and recorded

### Statistical analysis

Combined analysis of variance (ANOVA) was performed on all data to assess the amount of variability present among parents and their offspring using SAS (Statistical Analysis Software) version 9.4 (SAS Institute Inc., Cary, NC, USA). The mean performances of hybrids and paternal inbred lines were compared using the Least Significant Difference (LSD) method at a 5% level of significance. The general combining ability of parents and specific combining ability of hybrids were determined following Griffing’s method 2 model 1 using SAS software according to Zhang and Kang^51^.

### Estimation of heterosis

The relative heterosis (MP) and heterobeltiosis (BP) were determined and expressed as percentages^26^. The amount of heterosis was calculated using the differences between the mean of F_1_ hybrids and the mid parental value for a given characteristic^37^.

i. Mid-parent heterosis = [(F_1_ − MP)/MP] × 100 (relative heterosis).
  Where, F_1_ is the mean value of the F_1_, MP is the mean value of two parental involves in F_1_ i.e. (P_1_ + P_2_)/2.
  The significance of relative heterosis was tested using t-test^47^.
  $${\text{t }} = {\text{ F}}_{{1}} {\text{ij}}{-}{\text{MPij}}/({3}/{8}\sigma {\text{e}})^{{{1}/{2}}} ,$$
  where, F_1_ij is the mean of the ijth F_1_ cross, MPij is the mid-parent value for ijth cross, σ^2^e is the estimate of error variance.
ii. Better parent heterosis = [(F_1_ − BP)/BP] × 100 (heterobeltiosis).

Where, F_1_ is the mean value of the F_1_, BP is the mean value of better parent.

The significance of relative heterosis was tested using t-test^47^.

$${\text{t }} = {\text{ F}}_{{1}} {\text{ij}}{-}{\text{BPij}}/({1}/{2}\sigma^{{2}}_{{\text{e}}} )^{{{1}/{2}}} ,$$

where, F_1_ij is the mean of the ijth F_1_ cross, BPij is the Better value for ijth cross, σ^2^_e_ is the estimate of error variance.

## Result

### Qualitative variation

**Figure 1:**
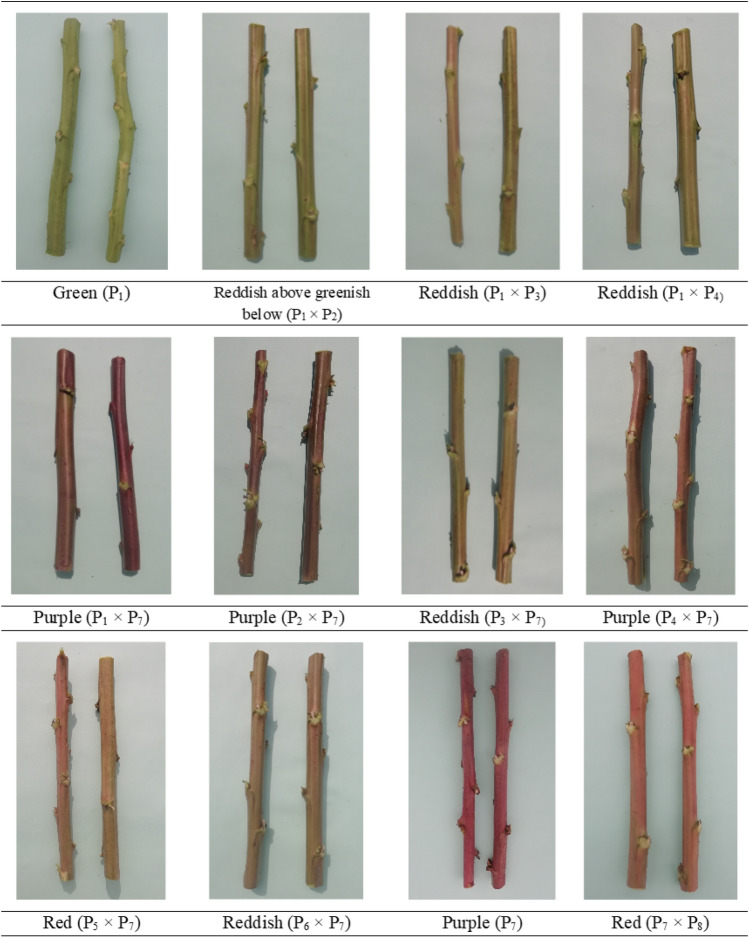
Photographs of the parents' and F_1_ population for stem coloration (70 DAS).

**Figure 2:**
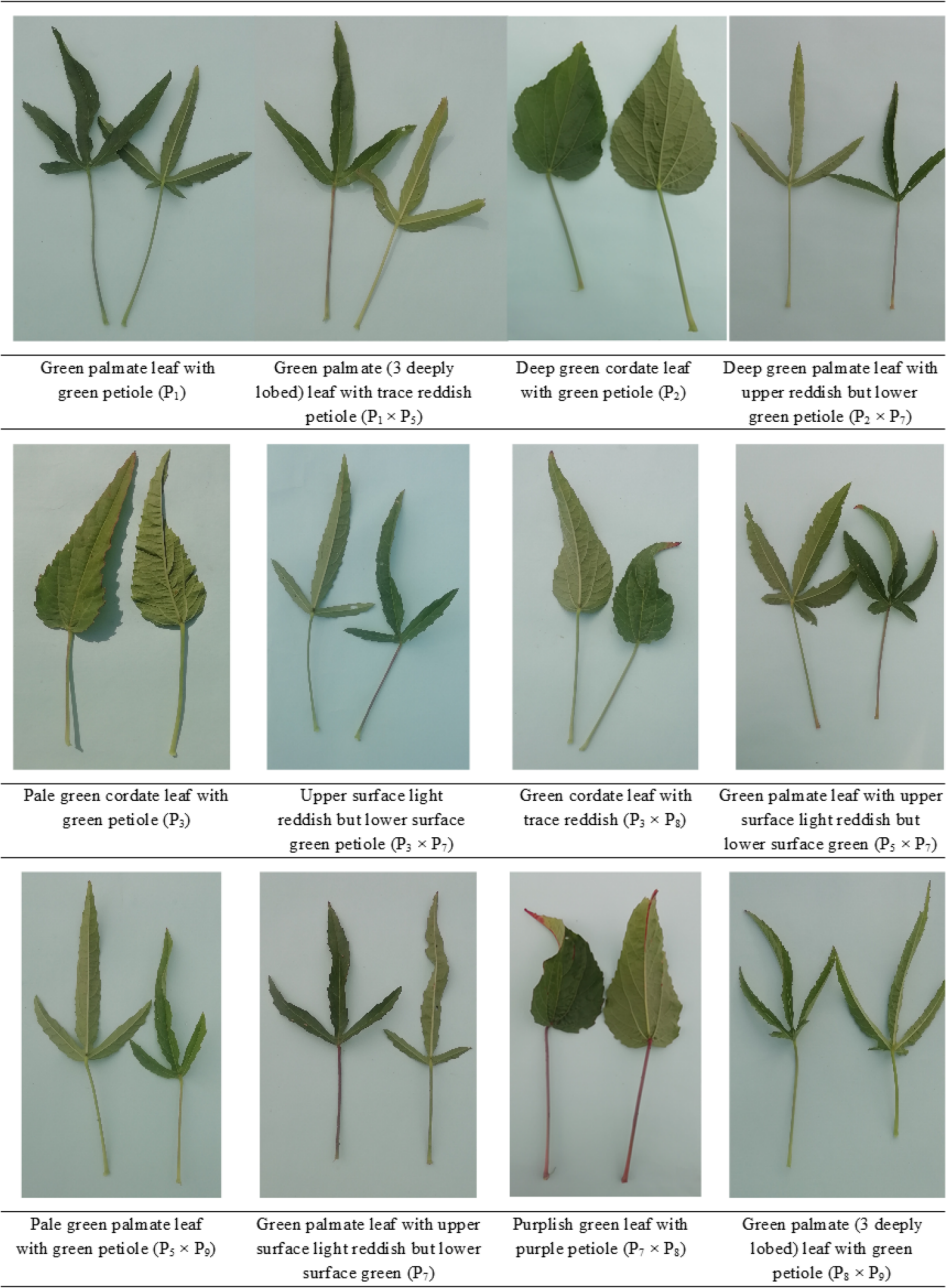
Photographs of the parents' and F_1_ population for leaf shapes and coloration.

**Figure 3:**
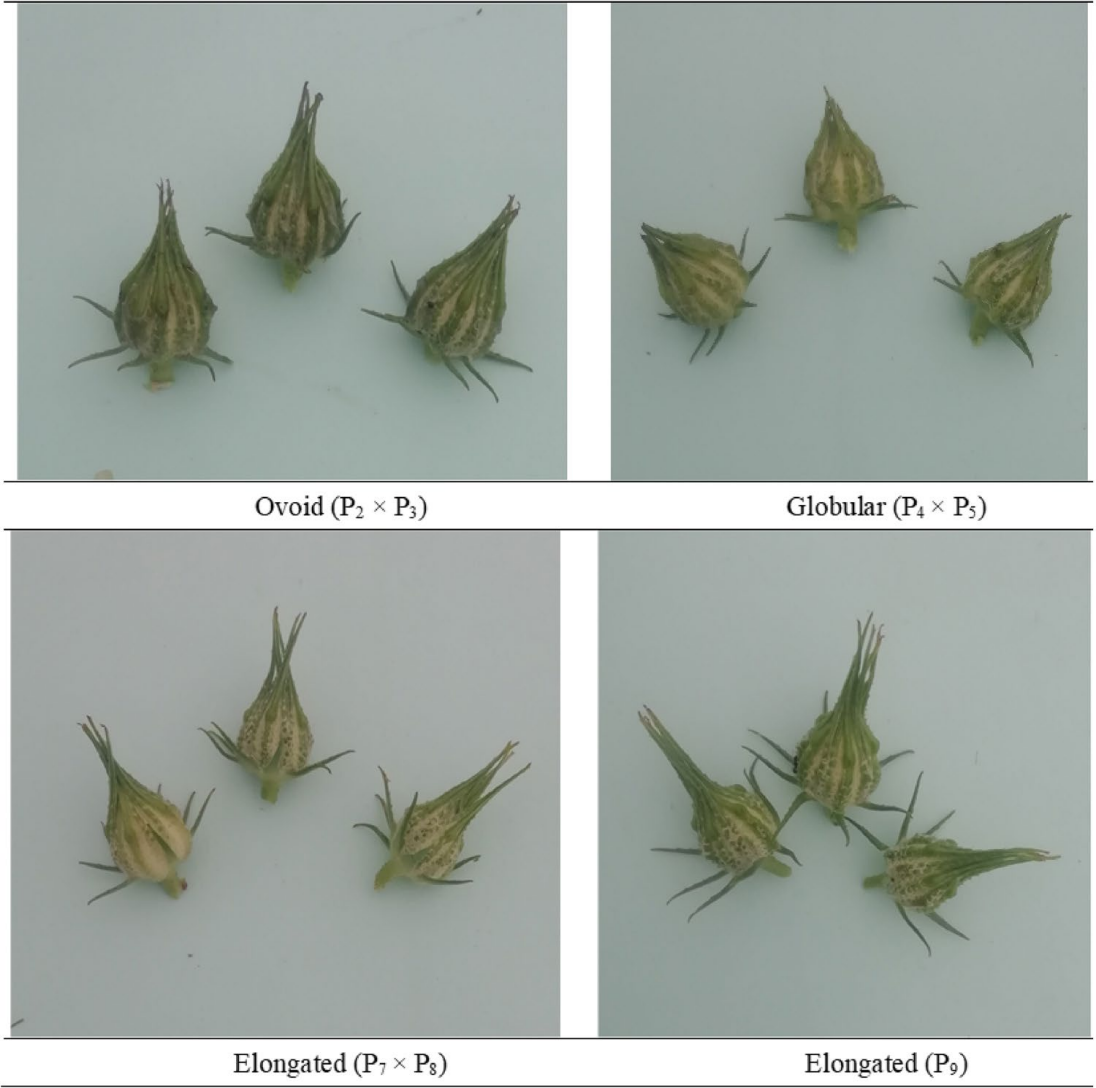
Photographs of the parents' and F_1_ population for pod shapes and colors.

Qualitative characteristics assessment provides information on highly diverse or uniform character, which can be quite different or very consistent (Table 3). Figures 1, 2 and 3 show the variation observed among the seven qualitative traits, revealing that plant, stem and leaf characteristics differed significantly.

**Table 3:** Characteristics of selected parents' growth stages and the F_1_ kenaf population.

Parents/hybrids	Stem color	Leaf shape	Leaf color (lamina)	Petiole color	Pod shape	Seed shape	Seed coat color
P_1_	Green	Palmate	Green	Green	Ovoid	Triangular	Ash gray
P_2_	Green	Cordate	Deep green	Green	Ovoid	Triangular	Ash gray
P_3_	Grp	Cordate	Pale green	Green	Ovoid	Triangular	Ash gray
P_4_	Grp	Cordate	Deep green	Green	Ovoid	Sub-reniform	Brownish
P_5_	Grp	Cordate	Green	Green	Globular	Triangular	Ash gray
P_6_	Grp	Cordate	Deep green	Green	Elongated	Triangular	Bfb
P_7_	Purple	Palmate	Green	UrLg	Ovoid	Triangular	Ash gray
P_8_	Grp	Cordate	Deep green	Green	Globular	Triangular	Ash gray
P_9_	Grp	Palmate	Green	Green	Elongated	Triangular	Ash gray
P_1_ × P_2_	RaGb	Palmate	Green	Green	Ovoid	Sub-reniform	Blackish
P_1_ × P_3_	Reddish	Palmate	Green	Green	Ovoid	Triangular	Blackish
P_1_ × P_4_	Reddish	Palmate	Green	Green	Ovoid	Triangular	Ash gray
P_1_ × P_5_	RaGb	Palmate	Green	Green	Ovoid	Triangular	Brownish
P_1_ × P_6_	RaGb	Palmate	Green	Green	Elongated	Sub-reniform	Blackish
P_1_ × P_7_	Purple	Palmate	Deep green	UrLg	Globular	Triangular	Ash gray
P_1_ × P_8_	RaGb	Palmate	Green	Green	Globular	Triangular	Ash gray
P_1_ × P_9_	RaGb	Palmate	Green	Green	Globular	Triangular	Ash gray
P_2_ × P_3_	Grp	Cordate	Deep green	Reddish	Globular	Sub-reniform	Blackish
P_2_ × P_4_	Grp	Cordate	Green	Green	Ovoid	Sub-reniform	Ash gray
P_2_ × P_5_	Grp	Cordate	Green	Green	Elongated	Triangular	Blackish
P_2_ × P_6_	Grp	Cordate	Green	Green	Ovoid	Triangular	Blackish
P_2_ × P_7_	Purple	Palmate	Deep green	UrLg	Globular	Triangular	Ash gray
P_2_ × P_8_	Grp	Cordate	Green	Green	Globular	Sub-reniform	Ash gray
P_2_ × P_9_	Green	Palmate	Deep green	Green	Ovoid	Triangular	Ash gray
P_3_ × P_4_	Green	Cordate	Green	Green	Globular	Triangular	Blackish
P_3_ × P_5_	Green	Cordate	Green	Green	Globular	Triangular	Blackish
P_3_ × P_6_	Grp	Cordate	Green	Green	Globular	Triangular	Ash gray
P_3_ × P_7_	Reddish	Palmate	Green	UrLg	Globular	Triangular	Bfb
P_3_ × P_8_	Grp	Cordate	Green	Green	Globular	Triangular	Ash gray
P_3_ × P_9_	Green	Palmate	Green	Green	Globular	Triangular	Blackish
P_4_ × P_5_	Green	Cordate	Deep green	Green	Globular	Triangular	Ash gray
P_4_ × P_6_	Green	Cordate	Deep green	Green	Globular	Triangular	Ash gray
P_4_ × P_7_	Purple	Palmate	Deep green	UrLg	Ovoid	Sub-reniform	Brownish
P_4_ × P_8_	Grp	Cordate	Green	Green	Ovoid	Triangular	Ash gray
P_4_ × P_9_	Grp	Palmate	Green	Green	Ovoid	Triangular	Blackish
P_5_ × P_6_	Grp	Cordate	Green	UrLg	Ovoid	Triangular	Brownish
P_5_ × P_7_	Red	Palmate	Deep green	UrLg	Globular	Triangular	Brownish
P_5_ × P_8_	Grp	Cordate	Green	Green	Ovoid	Triangular	Blackish
P_5_ × P_9_	Grp	Palmate	Pale green	Green	Ovoid	Triangular	Blackish
P_6_ × P_7_	Reddish	Palmate	Green	UrLg	Globular	Triangular	Ash gray
P_6_ × P_8_	Grp	Cordate	Deep green	Green	Globular	Triangular	Blackish
P_6_ × P_9_	Grp	Palmate	Green	Green	Globular	Triangular	Blackish
P_7_ × P_8_	Red	Cordate	Green	Purple	Ovoid	Triangular	Bfb
P_7_ × P_9_	Red	Palmate	Green	UrLg	Globular	Triangular	Brownish
P_8_ × P_9_	Grp	Palmate	Green	Green	Ovoid	Sub-reniform	Blackish

*Grp* Green with reddish patches, *RaGb* Reddish above greenish below, *UrLg* Upper surface light reddish but lower surface green, *Bfb* Black with few brownish.

### Parents

The selected parents' stem color, leaf shape, leaf color (lamina), and petiole color varied greatly. Two of the nine parents, P_1_ and P_2_, were green stem types with green petioles. Parent P_7_ had a light reddish upper surface but a green petiole on the lower surface with a purple stem. Otherwise, five parents (P_3_, P_4_, P_5_, P_6_, and P_8_) had green petioles with cordate leaves and green stems with reddish patches. Palmate leaf morphologies with green leaf colors were seen in parents P_1_, P_7_, and P_9_ (Table 3). Parents P_2_, P_4_, P_6_, and P_8_ had deep green leaves, whereas P_3_ had pale green leaves. The genotypes are further divided into three groups depending on pod shape: globular (round), elongated (pointed), and oval (egg-shaped). Genotypes differed in seed shape and seed coat colors. The parent P_4_ seed coat color were brownish, while the P_6_ seeds were black with a few brownish patches and the rest were ash gray.

### F_1_ generation

**Figure 4:**
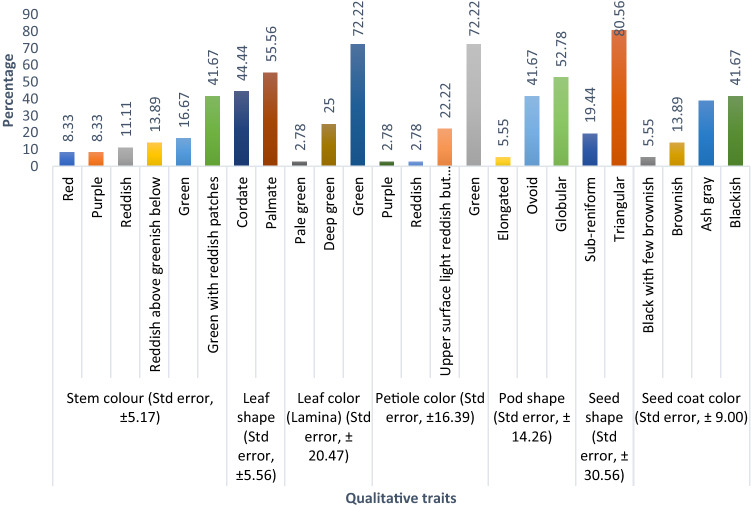
Qualitative variation in the kenaf F_1_ population (**a**) Stem color, (**b**) Leaf shape, (**c**) Leaf color (lamina), (**d**) Petiole color, (**e**) Pod shape, (**f**) Seed shape, and (**g**) Seed coat color.

The stem color showed a lot of variances with standard error (Sem, ± 5.17) (Fig. 1). The color variance for the green with reddish patches, green, reddish above greenish below, reddish, purple, and red stem colors was 41.67%, 16.67%, 13.89%, 11.11%, 8.33%, and 8.33%, respectively (Fig. 4a), from the 36 described kenaf F_1_ hybrids. Palmate leaves were found in 55.56% of F_1_ hybrids, whereas cordate leaves were found in 44.44% (Fig. 4b). The 36 F_1_ hybrids' leaf color (lamina) was diverse, falling into three categories: green, deep green, and pale green, which accounted for 72.22%, 25.00%, and 2.78% genotypes, respectively (Figs. 2 and 4c). Petiole color varied greatly among the hybrids investigated, with 72.22%, 22.22%, 2.78%, and 2.78% for green, upper surface light reddish but lower green, reddish, and purple, respectively (Fig. 4d). The form of the kenaf pod also showed a wide range of variation with standard error (Sem, ± 14.26). The genotypes are divided into three groups based on pod shape: globular (round) 52.78%; ovoid (egg-shaped) 41.67%; and elongated (pointed) 5.55% (Figs. 3 and 4e). Seed shape and seed coat color differed across the 36 F_1_ hybrids. Triangular seed shape was found in 80.56% of F_1_ hybrids, whereas sub-reniform seed shape was found in 19.44% (Fig. 4f). Blackish seeds were found in up to 41.67% of hybrids, ash gray seeds in 38.89%, brownish seeds in 13.89%, and black with few brownish color seeds in 5.55% (Fig. 4g).

### Variation among all genotypes for quantitative traits in pooled environments

Across the two environments, the combined analysis of variance for the 15 quantitative features among the nine parents and 36 crosses revealed significant differences (Table 4). There were significant differences (p ≤ 0.01) in environments and genotypes (parents and offspring) for all the variables studied, with exception of middle and top diameter, respectively. There were significant variations (p ≤ 0.01 or 0.05) for genotype by environment (G × E) except for plant height. Base diameter, core diameter, middle diameter, top diameter, number of nodes, days to 50% flowering, fresh stem weight with leaves and pod, fresh stem weight without leaves and pod, dry stick weight, dry fiber weight, number of pods per plant, number of seeds per pod, and 1000 seed weight were all shown to be highly significant differences (p ≤ 0.01) for G × E interactions. The CV% for yield and yield-related components ranges from 9.40 (days to 1st flowering) to 69.05 (nodes number), showing that the evaluated traits have a wide variability range.

**Table 4:** Combined analysis of variance was performed for 15 quantitative attributes of nine parents and their crosses over two environments.

Traits	Reps (environment)	Environments (E)	Genotypes (G)	G × E	Error	CV
DF	4	1	44	44	176	
PH	1315.93	47,673.09**	1814.32**	769.20	644.08	12.02
BD	53.89**	2644.85**	45.51**	28.09**	7.80	20.39
CD	60.42**	2716.06**	42.49**	30.47**	7.73	23.81
MD	128.33**	3.95	7.08**	4.75**	2.18	19.19
TD	42.75**	534.31**	1.24	1.59**	0.92	44.68
NN	2.27	5677.56**	10.16**	9.65**	2.28	69.05
DTFF	76.69**	258.13**	54.04**	19.53*	12.34	9.40
D50%F	122.47**	1946.76**	85.63**	59.30**	33.12	12.55
FW1	4918.52	18,630,479.18**	466,505.69**	488,941.97**	65,920.85	42.00
FW2	42,044.66**	471,710.49**	19,213.80**	10,701.47**	4361.17	30.88
DSW	3601.17**	18,465.68**	2344.96**	3071.62**	877.24	36.86
DFW	4.60	5968.64**	126.22**	102.29**	17.80	33.59
NF	3921.87**	80,067.83**	6548.07**	5281.92**	646.90	43.81
NS	12.62**	1966.57**	77.45**	49.12**	3.60	23.48
SW	0.97	2595.63**	23.57**	20.64**	1.46	13.52

*CV* coefficient of variation, *DF* degree of freedom, *PH* plant height, *BD* base diameter, *CD* core diameter, *MD* middle diameter, *TD* top diameter, *NN* number of nodes, *DTFF* days to first flowering, *D50%F* days to 50% flowering, *FW1* fresh stem weight with leaves and pod, *FW2* fresh stem weight without leaves and pod, *DSW* dry stick weight, *DFW* dry fiber weight, *NF* number of pods per plant, *NS* number of seeds per pod, *SW* 1000 seeds weight. **Highly significant at P ≤ 0.01 level, *Significant at P ≤ 0.05 level.

### Variation among all genotypes due to combining ability effects in pooled environments

Analysis of variance for combining ability using Griffing's (1956) technique was utilized for all analyzed qualities in the F_1_ combined data (Table 5) for both environments. General combining ability (GCA) is generally understood to be a consequence of additive gene effects and additive epistatic variance components. Specific combining ability (SCA), on the other hand, is a result of non-additive gene effects and the remaining epistatic variation (Matzinger et al., 1959). The mean squares analysis of variance results for the effects of combining ability are shown in Table 5. Significant differences (p ≤ 0.01 or 0.05) were observed for all studied traits for GCA except for top diameter and SCA except for plant height and top diameter, indicating the presence of both additive and non-additive gene action for the inheritance of the concerned characters. Except for plant height, and fresh stem weight without leaves and pod, significant differences (p ≤ 0.01 or 0.05) were reported for the interaction between GCA and environment. Except for plant height, top diameter, and days to first flowering, all traits showed significant variations (p ≤ 0.01 or 0.05) when SCA and environment interacted. Thus, the effects of non- additive genes in the traits interacted more with the environment. GCA and SCA ratios ranged from 0.54 (nodes number) to 8.29 (days to first flowering). For top diameter, nodes number, and fresh stem weight with leaves and pod, the ratio of GCA and SCA variances was found to be smaller than unity, indicating that non-additive gene action predominated. The estimated GCA/SCA was greater than unity for the other characters, indicating that additive gene effects predominated in their expression. In kenaf, Jianmin et al.^20^ and Heliyanto et al.^18^ found similar results.

**Table 5:** Mean squares of analysis of variance for combining ability of the 15 traits in pooled environments.

Traits	GCA	SCA	GCA*ENV	SCA*ENV	GCA/SCA
Plant height	5893.40**	835.22	800.55	766.61	7.06
Base diameter	64.04**	40.46**	19.28*	29.96**	1.58
Core diameter	53.48**	39.65**	22.09**	32.30**	1.35
Middle diameter	15.73**	5.19**	8.07**	4.01**	3.03
Top diameter	1.14	1.31	2.50**	1.40*	0.87
Number of nodes	5.94*	11.11**	8.55**	10.30**	0.53
Days to 1st flowering	188.16**	23.08**	36.03**	14.90	8.15
Days to 50% flowering	240.02**	49.77*	63.39	56.44*	4.82
Fresh stem weight with leaves and pod	470,219.89**	468,542.91**	576,405.65**	463,451.47**	0.99
Fresh stem weight without leaves and pod	45,353.95**	13,576.78**	4757.88	12,033.75**	3.34
Dry stick weight	5584.33**	1730.88**	2809.31**	2782.49**	3.23
Dry fiber weight	240.33**	104.98**	55.59**	111.88**	2.29
Number of pods per plant	9185.14**	5590.47**	4864.76**	5587.88**	1.64
Number of seeds per pod	51.31**	17.65**	47.78**	14.09**	2.91
1000 seeds weight	240.64**	41.05**	24.73**	55.12**	5.86

*GCA* general combining ability, *SCA* specific combining ability, *GCA × ENV* interaction of GCA and environment, *SCA×ENV* interaction of SCA and environment. *Significant at P ≤ 0.05, **highly significant at at P ≤ 0.01.

### Mean performance of genotypes over two environments

The mean comparison for all genotypes (parents and offspring) was presented in Table 6. Plant heights ranged from 219.58 to 297.85 cm, with hybrid P_3_ × P_7_ having higher mean value and P_2_ × P_9_ recorded the lowest. The base diameter ranged from 19.95 mm (P_2_ × P_9_) to 31.68 mm (P_2_ × P_3_), but the base diameters of P_1_ × P_9_ and P_7_ × P_8_ were comparable. The P_2_ × P_3_ had the largest core diameter (28.43 mm), while P_2_ × P_9_ had the smallest (16.83 mm). The middle diameter was 9.69 mm (P_2_) to 15.74 mm (P_5_ × P_8_), with the maximum and lowest top diameters of P_6_ × P_7_ and P_1_ × P_7_, respectively. The hybrid P_6_ × P_9_ had the most nodes (9.68), while P_2_ had the least (4.15). Days to first flowering ranged from 43 to 56.33 days in P_7_ × P_9_ and P_2_ × P_5_, respectively. Days to 50% flowering ranged from 51 to 69.17 days. The hybrid P_7_ × P_9_ was the first to mature, while parent P_2_ was the last to mature (Table 6).

**Table 6:** Mean performance of nine parents and their hybrids for 15 yield and yield contributing characters in kenaf.

Parents/Hybrids	PH	BD	CD	MD	TD	NN	DTFF	
P_1_	247.41i–l	25.74g–l	21.77j–n	12.17c–j	4.31b–h	6.91h–l	52.00b–i	
P_2_	230.44l	21.45n–q	17.89p–r	9.69k	4.47a–h	4.15p	54.17a–d	
P_3_	276.91a–g	24.70j–m	21.10l–p	12.49b–h	4.06e–h	4.80op	48.33i–n	
P_4_	280.59a–g	25.47g–m	21.88i–n	12.29b–j	4.31b–h	5.10m–p	52.67a–h	
P_5_	265.45c–i	24.61j–n	20.99l–p	12.23b–j	4.36a–h	7.38d–l	49.17g–m	
P_6_	255.22f–k	20.88p–q	17.15qr	10.95g–k	4.15c–h	5.10n–p	51.83b–j	
P_7_	278.07a–g	22.36m–q	19.06n–r	10.85h–k	4.10d–h	6.16l–o	45.17m–o	
P_8_	258.23d–k	21.40o–q	18.14o–r	11.57d–j	4.33b–h	6.82i–m	52.00b–i	
P_9_	271.53a–i	23.33k–p	19.53m–r	10.73i–k	3.77g–h	6.86h–l	49.50g–l	
$\overline{\mathrm{x} }$ Parents	262.65	23.33	19.72	11.44	4.21	5.92	50.54	
P_1_ × P_2_	252.56g–k	26.28e–k	22.35e–m	11.22f–k	3.66g–h	7.17e–l	52.67a–h	
P_1_ × P_3_	272.47a–i	29.22a–e	25.32a–f	12.65b–f	4.87a–f	9.23a–c	50.17d–l	
P_1_ × P_4_	264.25c–i	29.07a–f	25.09b–h	12.77b–f	4.06d–h	8.48a–i	50.00e–l	
P_1_ × P_5_	234.13jl	24.957i–m	21.40k–n	10.70i–k	4.19b–h	8.71a–f	53.83a–e	
P_1_ × P_6_	255.62e–k	25.47g–m	22.07g–n	11.64d–j	4.51a–h	8.53a–i	51.83b–j	
P_1_ × P_7_	285.13a–d	27.89c–i	24.65c–j	11.57d–j	3.45h	8.53a–i	50.33c–l	
P_1_ × P_8_	268.35b–i	26.35e–k	22.59d–m	12.09c–j	3.82f–h	7.78b–l	51.50b–k	
P_1_ × P_9_	251.86g–k	23.45k–p	19.72m–r	10.62jk	4.12d–h	6.35k–o	53.67a–f	
P_2_ × P_3_	244.52i–l	31.68a	28.43a	12.58b–g	4.72a–g	9.36ab	54.00a–e	
P_2_ × P_4_	277.81a–g	28.21b–h	24.48c–k	12.57b–g	4.10d–h	8.57a–h	55.33ab	
P_2_ × P_5_	252.45g–k	26.88d–j	23.73c–l	11.35f–k	3.46h–h	8.98a–d	56.33a	
P_2_ × P_6_	260.21d–j	25.79g–l	22.13g–n	11.17f–k	3.78g–h	8.01a–k	51.33b–k	
P_2_ × P_7_	269.95a–i	25.86g–l	22.02h–n	11.09f–k	3.68g–h	6.88h–l	46.33l–o	
P_2_ × P_8_	259.92d–j	24.48j–o	20.89l–p	11.51d–j	4.39a–h	7.47d–l	50.00e–l	
P_2_ × P_9_	219.58l	19.95q	16.83r	9.74k	4.26b–h	4.89n–p	52.67a–h	
P_3_ × P_4_	291.87a–c	29.25a–e	25.41a–e	13.09b–d	4.14c–h	8.67a–g	50.67c–k	
P_3_ × P_5_	285.98a–d	26.11e–l	22.62d–m	12.28b–j	4.24b–h	6.99g–l	48.67h–n	
P_3_ × P_6_	284.5a–e	25.99f–l	22.20f–n	12.24b–j	4.05e–h	7.24e–l	49.33g–l	
P_3_ × P_7_	297.85a	24.56j–o	20.78l–p	12.14c–j	4.36a–h	6.56j–n	45.00no	
P_3_ × P_8_	280.28a–g	27.01d–j	22.69d–m	12.31b–i	4.22b–h	8.87a–e	50.67c–k	
P_3_ × P_9_	270.78a–i	24.11j–o	20.67l–p	11.37e–k	4.60a–g	6.45j–o	50.67c–k	
P_4_ × P_5_	272.93a–i	29.16a–f	24.95b–i	13.88b	4.49a–h	7.99a–k	48.67h–n	
P_4_ × P_6_	272.75a–i	30.28a–c	26.48a–c	12.69b–f	4.15c–h	8.35a–i	47.50k–n	
P_4_ × P_7_	281.6a–f	25.54g–m	21.68j–n	12.73b–f	5.27ab	6.87h–l	47.50k–n	
P_4_ × P_8_	284.18a–e	29.90a–d	25.75a–d	13.19b–d	5.23a–c	8.35a–i	54.33a–c	
P_4_ × P_9_	276.71a–h	29.87a–d	25.86a–c	12.06c–j	4.91a–e	6.60j–n	49.67f–l	
P_5_ × P_6_	278.45a–g	23.65k–p	20.41m–p	11.85d–j	4.06e–h	7.11f–l	48.67h–n	
P_5_ × P_7_	280.43a–g	24.93i–m	21.37k–n	12.75b–f	4.30b–h	7.10f–l	45.00no	
P_5_ × P_8_	294.45ab	31.15ab	27.80ab	15.74a	5.15a–d	8.13a–j	51.50b–k	
P_5_ × P_9_	248.78h–k	23.03l–q	19.82m–r	11.56d–j	3.77g–h	7.63c–l	47.83j–n	
P_6_ × P_7_	271.49a–i	24.85i–m	21.62j–n	12.33b–i	5.44a	7.18e–l	47.67k–n	
P_6_ × P_8_	263.88c–i	25.21h–m	21.37k–n	10.82h–k	4.53a–h	6.91h–l	52.83a–g	
P_6_ × P_9_	260.09d–j	26.13e–l	22.40e–m	11.89d–j	4.91a–f	9.68a	48.50i–n	
P_7 ×_ P_8_	286.03a–d	23.45k–p	20.08m–q	11.28f–k	4.09d–h	6.09l–o	45.17m–o	
P_7_ × P_9_	296.17ab	28.52a–g	25.20b–g	13.03b–e	4.28b–h	7.47d–l	43.00o	
P_8_ × P_9_	261.7d–j	24.88i–m	21.54j–n	13.67b–c	4.31b–h	9.09a–d	48.33i–n	
$\overline{\mathrm{x} }$ Hybrids	269.71	26.47	22.84	12.11	4.32	7.73	50.03	
Overall Mean	268.30	25.85	22.22	11.98	4.30	7.37	50.13	
Std Dev	32.26	5.27	5.29	2.30	1.92	5.09	4.71	
Std Error	1.96	0.32	0.32	0.14	0.12	0.31	0.29	
EMS	644.08	7.80	7.73	2.18	0.92	2.28	12.34	
LSD (5%)	28.92	3.18	3.17	1.68	1.09	1.72	4.00	

Parents/hybrids	D50%F	FW1	FW2	DSW	DFW	NF	NS	SW
P_1_	60.67b–j	1127.20k–r	328.89e–l	93.78g–o	26.71e–i	86.83o–t	26.08b–f	34.18ab
P_2_	69.17a	670.50tu	225.00o	74.56no	17.56no	50.82u	20.08l–o	33.78a–d
P_3_	57.67c–m	962.00n–t	331.95d–k	123.24a–h	22.82h–m	70.83s–u	26.65a–e	31.92g–m
P_4_	63.83a–e	1118.00l–r	351.42b–i	90.62h–o	24.78g–k	67.56tu	26.02b–f	30.50n–s
P_5_	58.00c–m	818.10s–u	247.97m–o	79.60l–o	19.49m–o	92.84n–t	28.76a	30.33p–s
P_6_	64.00a–d	929.30o–t	222.41o	71.35o	17.65no	97.54m–s	25.73b–g	33.79a–d
P_7_	55.67i–n	937.80n–t	258.77k–o	96.27f–o	16.30o	121.94h–m	20.59k–n	32.65c–i
P_8_	60.50b–j	911.30p–t	287.22h–o	95.06g–o	23.59h–m	83.87p–t	27.36ab	33.04b–g
P_9_	55.83i–n	1102.30l–s	255.87l–o	81.46k–o	19.17m–o	106.78k–p	27.79ab	32.47d–j
$\overline{\mathrm{x} }$ Parents	60.59	952.95	278.83	89.55	20.89	86.56	25.45	32.52
P_1_ × P_2_	59.83c–k	1214.50f–o	328.52e–l	97.78f–o	25.96f–i	115.30i–o	15.08s	33.90a–c
P_1_ × P_3_	59.83c–k	1526.80a–d	379.58a–f	122.30a–i	31.54b–d	136.92d–j	25.95b–f	32.79c–i
P_1_ × P_4_	57.50d–n	1490.30b–g	410.69a–c	152.93a	31.34b–e	156.96b–f	25.85b–f	33.36b–f
P_1_ × P_5_	61.50b–i	1112.80l–r	272.33j–o	103.16d–n	21.11j–n	95.92m–t	22.57h–k	26.20x
P_1_ × P_6_	61.00b–j	1362.20c–l	316.11e–m	108.05c–n	25.12g–k	123.14g–m	20.03m–o	30.70m–r
P_1_ × P_7_	56.83f–n	1483.90b–h	350.83b–i	111.67c–m	27.42c–h	162.83b–d	20.69k–n	31.83g–o
P_1_ × P_8_	58.83c–m	1211.10f–o	323.43e–l	109.12c–m	27.25d–h	151.15b–g	25.79b–g	32.65c–i
P_1_ × P_9_	62.83a–f	1044.80m–s	312.56e–m	84.37j–o	24.62g–k	144.50b–h	24.54e–h	31.13j–q
P_2_ × P_3_	61.33b–j	1521.70a–e	414.45a–c	110.00c–m	38.74a	75.88q–u	16.45q–s	32.91b–h
P_2_ × P_4_	64.17a–c	1230.00e–n	386.11a–e	121.12a–i	31.46b–e	109.50j–p	18.42o–q	32.44d–j
P_2_ × P_5_	66.50a	1192.90h–p	322.08e–m	104.17c–n	29.03b–g	101.67m–r	20.25l–o	31.57h–p
P_2_ × P_6_	59.00c–l	1071.90l–s	331.67e–k	90.58h–o	26.04f–i	96.58m–s	24.96c–g	32.00f–m
P_2_ × P_7_	52.33n	1301.60d–m	318.10e–m	107.78c–n	26.00f–i	131.35e–l	20.39l–o	30.85l–q
P_2_ × P_8_	59.33c–l	1198.20g–p	303.06g–n	95.70g–o	25.91f–j	140.24d–i	15.74rs	35.05a
P_2_ × P_9_	59.83c–k	584.30u	229.22no	78.82mo	18.94m–o	83.91p–t	17.81p–r	33.67b–e
P_3_ × P_4_	57.67c–m	1573.90a–d	415.19a–c	133.33a–e	29.93b–f	98.61m–s	25.67b–g	29.40r–u
P_3_ × P_5_	59.50c–l	1478.80b–hh	383.71a–e	130.00a–f	25.89f–j	123.69g–m	26.56b–e	31.49i–p
P_3_ × P_6_	59.00c–l	1101.90l–s	305.56f–m	114.54b–k	22.86h–m	136.07d–j	24.18f–i	30.98k–q
P_3_ × P_7_	51.00n	1137.00k–r	353.02b–h	126.30a–g	24.88g–k	105.17l–p	20.95k–n	31.69g–p
P_3_ × P_8_	58.17c–m	1413.90b–k	343.33c–j	130.00a–f	24.78g–k	134.39d–k	23.68g–j	28.35u–v
P_3_ × P_9_	58.33c–m	887.90q–t	290.00h–o	105.31c–n	22.29i–n	106.08k–p	26.72a–d	29.90q–t
P_4_ × P_5_	59.33c–l	1660.40ab	367.50b–g	116.79b–j	28.76b–g	120.28h–n	26.68a–e	28.72t–v
P_4_ × P_6_	55.67i–n	1786.50a	419.35ab	134.46a–d	28.87b–g	172.30b	24.98c–g	32.11f–l
P_4_ × P_7_	58.67c–m	1174.30i–q	303.52g–n	112.92c–l	23.47h–m	75.45r–u	19.26n–p	27.55v–x
P_4_ × P_8_	60.50b–j	1360.70c–l	403.78a–d	134.09a–d	29.05b–g	96.00m–t	26.86a–c	29.78q–t
P_4_ × P_9_	56.33f–n	1627.00a–c	449.96a	147.69a	32.11bc	100.78m–r	27.30ab	32.31e–k
P_5_ × P_6_	57.67c–m	850.90r–u	266.85k–o	95.37g–o	22.29i–n	104.46l–q	26.47b–e	29.18s–u
P_5_ × P_7_	53.17ln	1209.40f–o	329.35e–l	92.22h–o	19.73l–o	160.25b–e	26.78a–d	30.72m–r
P_5_ × P_8_	56.17 g–n	1655.30ab	419.49ab	137.78a–c	32.81b	134.25d–k	22.22i–l	26.95wx
P_5_ × P_9_	56.00 h–n	1206.70g–o	277.08i–o	103.78d–n	25.14f–k	130.72f–l	24.67d–h	30.43p–s
P_6_ × P_7_	57.33e–n	1088.90l–s	270.83j–o	88.89i–o	20.36k–o	144.31b–h	22.63h–k	31.87g–n
P_6_ × P_8_	62.67a–g	1435.10b–j	294.83g–o	90.72h–o	22.16i–n	158.92b–f	26.28b–f	31.82g–o
P_6_ × P_9_	62.50b–h	1463.20b–i	305.50f–m	100.12e–n	24.51g–l	170.63bc	25.70b–g	30.46o–s
P_7 ×_ P_8_	53.33 k–n	1149.60j–q	313.34e–m	109.30c–m	22.05i–n	113.99i–o	19.18n–p	28.03u–w
P_7_ × P_9_	51.00n	1652.40a–c	348.28b–i	132.68a–e	25.56f–j	217.15a	21.82j–m	30.07q–t
P_8_ × P_9_	54.83j–n	1501.90a–f	296.76g–o	115.65b–j	25.03g–k	142.45c–i	27.27ab	30.33p–s
$\overline{\mathrm{x} }$ Hybrids	58.32	1304.52	337.67	112.49	26.19	126.99	23.07	30.92
OA Mean	58.77	1234.20	325.90	107.90	25.13	118.91	23.54	31.24
Std Dev	7.38	518.40	100.62	39.78	8.44	52.10	5.53	4.23
Std Error	0.45	31.55	6.12	2.42	0.51	3.17	0.34	0.26
EMS	33.12	65,920.85	4361.17	877.24	17.80	646.90	3.60	1.46
LSD5%	6.56	292.55	75.25	33.75	4.81	28.98	2.16	1.38

*PH* plant height (cm), *BD* base diameter (mm), *CD* core diameter (mm), *MD* middle diameter (mm), *TD* top diameter (mm), *NN* number of nodes, *DTFF* days to 1st flowering, *Std Dev* standard deviation, *EMS* error means square, *LSD* Least significant difference, *D50%F* Days to 50% flowering, *FW1* fresh stem weight with leaves and pod (g), *FW2* fresh stem weight without leaves and pod (g), *DSW* dry stick weight (g), *DFW* dry fiber weight (g), *NF* number of pods per plant, *NS* number of seeds per pod, *SW* 1000 seeds weight (g), *OA mean* overall mean. Means with the same letter in each letter in each column are not significantly different at 5% probability level.

Fresh stem weight with leaves and pods ranged from 584.30 g (P_2_ × P_9_) to 1786.50 g (P_4_ × P_6_), with the highest and lowest fresh stem weight without leaves and pods being 449.96 g (P_4_ × P_9_) and 222.41 g (P_6_), respectively. The dry stick weight ranged from 71.35 to 152.93 g, with P_1_ × P_4_ having the highest mean value (152.93 g), followed by P_4_ × P_9_ (147.69 g), and P_6_ having the lowest mean value (71.35 g). The hybrid P_2_ × P_3_ yielded its most dry fiber weight per plant (38.74 g), whereas P_7_ yielded the least (16.3 g). Aside from that, the hybrid P_7_ × P_9_ produced the most pods per plant (217.15), whereas P_2_ had the least (50.82). The number of seeds per pod ranged from 15.08 to 28.76. Parent P_5_ had the most seeds per pod, while P_1_ × P_2_ and P_2_ × P_8_ contained the fewest. The weight of 1000 seeds varied from 26.20 to 35.05 g. The hybrid P_2_ × P_8_ had the highest 1000 seed weight (35.05 g), while hybrid P_1_ × P_5_ had the lowest 1000 seed weight (26.20 g) (Table 6).

Plant height, base diameter, core diameter, middle diameter, top diameter, number of nodes, fresh stem weight with leaves and pod, fresh stem weight without leaves and pod, dry stick weight, dry fiber weight, and pods number per plant are all higher than parental mean. In comparison to the hybrid mean, the parental mean has somewhat longer days to first flowering and days to 50% flowering. Similarly, the parental mean had more seeds per pod and 1000 seed weight than the hybrid means. The fact that the hybrid mean has a lower weight per 1000 seeds than the parental mean is advantageous because it allows for smaller seed sizes. The largest standard deviation (SD) value was observed for fresh stem weight with leaves and pod (518.40) with standard error (Sem, ± 31.55), while the lowest was for top diameter (SD, 1.92; Sem, ± 0.12) (Table 6). The standard error (SE) indicates consistency of the average values, lower SE values suggest that the sample mean is a more precise depiction of the true population mean.

### Combining ability effects on genotypes (parents and offspring)

#### General combining ability effects on genotypes (parents and offspring) in pooled environment

Estimates of GCA effects of individual parental genotypes in the F_1_ generation were found to be statistically significant or highly significant for most traits studied. Plant height in a taller stature combination should have a positive GCA effect, while the nodes number should have a negative GCA effect. In the pooled data, the parent P_7_ had the maximum plant height (11.99 cm) and the lowest negative GCA values for nodes number (− 0.39), indicating that they were good general combiners for quality fiber yield and might be used in future breeding efforts. In terms of base diameter (2.32), core diameter (2.12), middle diameter (0.80), fresh stem weight with leaves and pod (176.17), fresh stem weight without leaves and pod (59.86), dry stick weight (16.57), and dry fiber weight (3.53), parent P_4_ had the greatest positively significant GCA effect. Excluding the P_4_, parent P_1_ (2.00) and P_3_ (1.89) were also shown to be positive and significant general combiners for dry fiber weight, with parent P_3_ having a positive and highly significant GCA effect (15.09) for dry stick weight.

The parents P_1_ (9.02), P_6_ (12.76), and P_7_ (17.57) had much higher seed output, which is a critical element in determining the pods number per plant. The parent P_2_ had the most positively significant GCA effect, with values of 2.32 and 3.17 for days to 1st flowering and days to 50% flowering, respectively. With − 3.74 and − 3.71, respectively, for days to first flowering and days to 50% flowering, parent P_7_ had the smallest GCA effect (Table 7). The parent P_2_ showed the lowest negative and highly significant GCA effect, with values of − 17.73, − 140.47, − 18.64, and − 4.50 for plant height, fresh stem weight with leaves and pod, pods number per plant, and seeds number per pod, respectively. For base diameter (− 2.34), core diameter (− 2.49), and fresh stem weight without leaves and pods (− 64.18), the parent P_9_ exhibited the lowest negative GCA values. A negative GCA effect is desired for 1000 seed weight to give a smaller seed size combination. The parent P_5_ had the most desirable 1000 seed weight with a negative GCA effect (− 1.63) and the highest positive (1.36) for seeds number per pod, both of which are highly significant, indicating that they were good general combiners for reduced seed sizes and the highest seeds number per pod in future breeding programs.

**Table 7:** Estimates of general combining ability effect (GCA) for 15 morphological characters of kenaf.

Traits	P_1_	P_2_	P_3_	P_4_	P_5_	P_6_	P_7_	P_8_	P_9_	LSD (gi–gj)	
										5%	1%
PH	− 10.34**	− 17.73**	8.11*	8.22**	− 1.31	− 3.22	11.99**	2.03	2.24	4.11	5.43
BD	0.68	− 0.43	0.96**	2.32**	0.22	− 0.69*	− 0.58	− 0.14	− 2.34*	0.45	0.60
CD	0.60	− 0.32	0.92**	2.12**	0.36	− 0.65	− 0.42	− 0.11	− 2.49*	0.45	0.59
MD	− 0.11	− 0.74**	0.44*	0.80**	0.53**	− 0.21	− 0.01	0.45*	− 1.15*	0.24	0.32
TD	− 0.10	− 0.13	0.09	0.24*	.00	0.12	0.06	0.18	− 0.46	0.16	0.20
NN	0.49**	− 0.33	− 0.03	0.07	0.38*	− 0.01	− 0.39*	0.28	− 0.47	0.24	0.32
DTFF	1.50**	2.32**	− 0.51	0.69	− 0.24	− 0.01	− 3.74**	0.63	− 0.64	0.57	0.75
D50%F	1.25	3.17**	− 0.51	1.07	0.01	1.55*	− 3.71**	− 0.09	− 2.74	0.93	1.23
FW1	45.42	− 140.47**	33.15	176.17**	− 17.97	− 16.56	− 11.68	49.76	− 117.80	41.59	54.89
FW2	13.76	− 10.66	31.66**	59.86**	− 6.01	− 22.27**	− 8.70	6.54	− 64.18**	10.70	14.12
DSW	2.24	− 8.84*	15.09**	16.57**	− 0.89	− 7.89*	2.00	5.47	− 23.76*	4.80	6.33
DFW	2.00**	1.04*	1.89**	3.53**	− 0.19	− 1.66**	− 2.15**	0.95	− 5.41**	0.68	0.90
NF	9.02**	− 18.64**	− 9.33**	− 8.74**	− 0.38	12.76**	17.57**	7.09*	− 9.34	4.12	5.44
NS	− 0.56*	− 4.50**	0.42	0.75**	1.36**	0.72**	− 2.36**	0.27	3.91**	0.31	0.41
SW	0.65**	1.47**	− 0.22	− 0.65**	− 1.63**	0.26	− 0.54**	− 0.43**	1.09*	0.20	0.26

*LSD* least significant difference, *PH* plant height, *BD* base diameter, *CD* core diameter, *MD* middle diameter, *TD* top diameter, *NN* number of nodes, *DTFF* days to 1st flowering, *D50%F* days to 50% flowering, *FW1* fresh stem weight with leaves and pod, *FW2* fresh stem weight without leaves and pod, *DSW* dry stick weight, *DFW* dry fiber weight, *NF* number of pods per plant, *NS* number of seeds per pod, *SW* 1000 seeds weight. **Highly significant at P ≤ 0.01 level, *Significant at P ≤ 0.05 level.

### Specific combining ability effects on hybrids across the environment

The effects of specific combining ability on hybrids in various contexts in pooled environments are shown in Table 8. SCA effects were detected in all 36 hybrids studied, with positive (desired direction) SCA influences on plant height in 19 of them. The hybrid with significant and beneficial SCA effects were produced by the cross P_5_ × P_8_ (24.44), which was evaluated as a good specific combiner for tallness. The hybrids P_1_ × P_5_ (− 23.51) and P_2_ × P_9_ (− 37.62) were identified as the worst specific combiners for this trait, as shown by the significant negative SCA effects for plant height. The SCA effects on the base diameter ranged from − 6.33 to 5.47. Three of the 16 positive SCA effects for base diameter on the were found to be best specific combiners with highly significant (P_2_ × P_3_, P_4_ × P_6_ and P_5_ × P_8_), while two were found to be good specific combiners with significant (P_1_ × P_7_ and P_7_ × P_9_). Other 20 crosses had negative SCA effects, including one (P_2_ × P_9_) with a highly significant SCA value of − 6.33.

Out of 36 cross combinations, five showed significant positive SCA values for kenaf core diameter, indicating heterotic performance over the mean of their parents. The cross P_2_ × P_3_ had the highest positive SCA effect (5.81), followed by P_5_ × P_8_, P_7_ × P_9_ and P_4_ × P_6_, indicating that they were the best specific combiners for the trait. The cross P_2_ × P_9_ had the most negative and highly significant SCA effect (− 5.86) and was considered the worst specific combiner for core diameter. The SCA effects for mid-diameter stem varied from − 1.60 to 2.88. The hybrid P_5_ × P_8_ had the best SCA effects with highly significant, while another 18 crosses had positive but insignificant SCA values, implying that they could be used as average specific combiners. Other 17 crossings had negative SCA effects, with one (P_1_ × P_5_) being highly significant (− 1.60) and another (P_6_ × P_8_) being significant (− 1.31). These are considered the worst specific combiners for mid-diameter (Table 8).

The SCA effects on top diameter ranged from − 0.75 to 1.10. Since four crossings revealed significant positive SCA effects, one of which was highly significant, P_2_ × P_9_, P_4_ × P_7_ and P_5_ × P_8_ and P_6_ × P_7_ were chosen as good specific combiners for this trait. The SCA values of another 15 crosses were positive but not significant, indicating that they may be regarded as average specific combiners for top diameter. SCA impacts ranged from − 4.15 to 2.39 for a given number of nodes. Hybrid P_2_ × P_9_ (− 4.15) had the highest negative and highly significant SCA estimate, followed by P_3_ × P_9_ (− 2.86), P_4_ × P_9_ (− 2.71), and P_1_ × P_9_ (− 2.38), with one negative significant P_7_ × P_8_ (− 1.13), showing that these hybrids had good SCA for lower branch stem and improved fiber yield. Three of the 21 positive SCA effects, on the other hand, were found to be highly significant, and three were found to be significant, therefore they were classified as poor specific combiners for nodes number.

The SCA effects ranged from − 3.32 to 4.10 for days to first flowering (Table 8). The cross (P_2_ × P_5_) was positively highly significant, with two more significant hybrids (P_4_ × P_8_ and P_1_ × P_9_), showing that these hybrids had good SCA for this trait. In contrast, the offspring P_4_ × P_6_ exhibited the highest negative and significant SCA estimate, followed by P_2_ × P_8_, all of whom were considered poor specific combiners for the days to first flowering trait. SCA impacts ranged from − 5.71 to 7.44 for days to 50% flowering. The cross P_6_ × P_9_ of the 15 positive SCA effects, which displayed a good specific combining capacity for this characteristic, had the most significant SCA effects followed by P_2_ × P_5_. Instead, the crosses P_2_ × P_6_, P_2_ × P_7_ and P_4_ × P_6_ had the most negative and significant SCA estimates, indicating that they were poor specific combiners for days to 50% flowering.

Four crosses viz. P_2_ × P_3_, P_4_ × P_5_, P_4_ × P_6_ and P_5_ × P_8_ were the best specific combiners for fresh stem weight with leaves and pod trait, with highly significant positive SCA effects. Five more crossings of the 20 positive SCA effects viz. P_1_ × P_3_, P_1_ × P_7_, P_2_ × P_7_, P_3_ × P_5_ and P_7_ × P_9_ produced positive and significant SCA effects, making them good specific combiners. Four of the remaining 16 crosses had significant SCA values, indicating negative SCA effects. As a result, they were regarded as poor specific combiners for fresh stem weight with leaves and pod characteristics.

Two crosses, P_5_ × P_8_ (98.91) and P_2_ × P_3_ (73.40) had highly significant positive SCA effects, whereas one cross, P_4_ × P_6_ (61.71), had significant positive SCA effects, indicating that these hybrids had good specific combining ability for fresh stem weight without leaves and pod trait. Another 18 crossings had positive, but minor, SCA impacts and might be classified as average specific combiners. The remaining 15 crosses had negative SCA effects, with three (P_1_ × P_5_, P_2_ × P_9_, and P_4_ × P_7_) having significant SCA values and so being classified as poor specific combiners for fresh stem weight without leaves and pod characteristic.

In kenaf, 18 cross pairings had positive SCA effects, while the other 18 had negative for the stick weight per plant, SCA impacts ranging from − 15.27 to 36.25. Hybrid P_7_ × P_9_ had the largest significant positive SCA impact (36.25), followed by P_1_ × P_4_ (28.89) and P_5_ × P_8_ (27.97), implying that it was a good specific combiner for the trait of stick weight per plant. Three crosses (P_1_ × P_9_, P_2_ × P_9_ and P_5_ × P_7_) having negatively significant SCA values, making them poor specific combiners for dry stick weight.

Two crosses, P_2_ × P_3_ (11.24) and P_5_ × P_8_ (7.47) showed highly significant positive SCA effects, while one cross, P_2_ × P_5_ (3.60) showed significant positive SCA effects, implying better fiber yielding hybrids than their parents' mean and considered best specific combiners for increased fiber weight per plant of kenaf. The remaining 17 crosses, on the other hand, had positive SCA findings, but 16 had negative SCA outcomes. Crosses P_1_ × P_5_ (− 5.28) and P_2_ × P_9_ (− 10.37) were the worst specific combiners for the trait in question, with highly significant negative values.

The SCA effects on the number of pods per plant ranged from − 49.49 to 63.49. (Table 8). The cross combinations produced 21 positive SCA effects, with 11 of them having significant positive SCA values for the number of pods per plant. The crosses P_2_ × P_3_ (7.20), P_3_ × P_4_ (4.70), and P_2_ × P_4_ (3.32) had highly significant positive SCA effects, but P_4_ × P_6_ (− 3.43), P_1_ × P_3_ (− 2.18), and P_1_ × P_5_ (− 1.05) had highly significant negative SCA effects.

SCA impacts ranged from − 4.01 to 4.87 for the quantity of seeds per pod (Table 8). Sixteen of the cross combinations had positive SCA effects, with six (P_1_ × P_3_, P_1_ × P_8_, P_2_ × P_6_, P_2_ × P_7_, P_4_ × P_8_ and P_5_ × P_7_) having highly significant positive SCA values and two (P_1_ × P_4_ and P_6_ × P_8_) having significant positive SCA values considered as good specific combiners for the number of seeds per pod characteristic. The remaining 20 crosses had negative SCA effects, with 12 of them having highly significant negative SCA effects and were considered the poorest specific combiners for the trait under consideration.

For the character 1000 seeds weight, the SCA effects ranged from − 4.20 to 2.64 (Table 8). Hybrids P_1_ × P_5_, P_1_ × P_6_, P_2_ × P_7_, P_3_ × P_8_, P_4_ × P_7_, P_5_ × P_8_, and P_7_ × P_8_, all exhibited highly significant negative SCA effects, whilst P_3_ × P_9_, P_3_ × P_4_, and P_2_ × P_6_ had significant negative SCA effects, indicating that these hybrids had good specific combining capacity for smaller seed size. Seven more crossings exhibited negative but minor SCA effects, indicating that they are average specific combiners. The remaining 19 crosses had positive SCA effects, with five having highly significant SCA values and three having significant SCA values that were considered poor specific combiners for the 1000 seed weight trait.

**Table 8:** Estimates of specific combining ability (SCA) effects for 15 different morphological characters of kenaf.

Hybrids	PH	BD	CD	MD	TD	NN	DTFF	D50%F	FW1	FW2	DSW	DFW	NF	NS	SW
P_1_ × P_2_	11.33	0.36	0.06	0.20	− 0.35	− 0.31	− 1.31	− 3.16	89.44	5.37	− 0.85	− 1.66	8.80	− 3.73**	0.40
P_1_ × P_3_	5.4	1.91	1.78	0.44	0.64	1.45*	− 0.97	0.52	228.07*	14.12	− 0.26	3.07	21.11*	2.20**	0.98*
P_1_ × P_4_	− 2.92	0.40	0.36	0.20	− 0.32	0.59	− 2.34	− 3.39	48.58	17.03	28.89*	1.23	40.57**	1.78*	1.98**
P_1_ × P_5_	− 23.51*	− 1.61	− 1.57	− 1.60**	0.05	0.52	2.42	1.67	− 134.78	− 55.46*	− 3.42	− 5.28**	− 28.84**	− 2.11**	− 4.20**
P_1_ × P_6_	− 0.11	− 0.19	0.11	0.08	0.24	0.73	0.19	− 0.38	113.25	4.57	8.48	0.20	− 14.76	− 4.01**	− 1.59**
P_1_ × P_7_	14.19	2.12*	2.46*	− 0.19	− 0.75*	1.11	2.42	0.71	230.03*	25.72	2.20	2.99	20.13*	− 0.28	0.34
P_1_ × P_8_	7.36	0.14	0.09	− 0.14	− 0.51	− 0.31	− 0.78	− 0.91	− 104.18	− 16.92	− 3.82	− 0.28	18.92*	2.20**	1.06*
P_1_ × P_9_	− 10.54	− 1.84	− 1.84	0.50	0.72	− 2.38**	1.51	5.34	− 286.68	24.25	− 15.27	1.58	− 18.62	0.63	− 0.48
P_2_ × P_3_	− 15.15	5.47**	5.81**	1.00	0.52	2.39**	2.04	0.09	408.87**	73.40**	− 1.48	11.24**	− 12.26	− 3.35**	0.29
P_2_ × P_4_	18.03	0.65	0.67	0.64	− 0.25	1.50**	2.18	1.35	− 25.82	16.87	8.16	2.32	20.77*	− 1.71*	0.24
P_2_ × P_5_	2.20	1.42	1.68	− 0.32	− 0.66	1.60**	4.10**	4.74*	131.24	18.71	8.67	3.60*	4.58	− 0.49	0.35
P_2_ × P_6_	11.87	1.23	1.09	0.24	− 0.46	1.02	− 1.12	− 4.30*	8.79	44.55	2.09	2.08	− 13.65	4.87**	− 1.11*
P_2_ × P_7_	6.40	1.20	0.75	− 0.03	− 0.49	0.27	− 2.40	− 5.71**	233.61*	17.41	9.39	2.54	16.32	3.37**	− 1.46**
P_2_ × P_8_	6.33	− 0.63	− 0.70	− 0.07	0.10	0.19	− 3.09*	− 2.33	68.77	− 12.87	− 6.17	− 0.66	35.68**	− 3.91**	2.64**
P_2_ × P_9_	− 37.62**	− 6.33**	− 5.86**	− 0.95	1.10*	− 4.15**	0.22	5.07	− 646.22**	− 89.71*	− 6.81	− 10.37**	− 32.23*	− 0.26	− 0.81
P_3_ × P_4_	6.26	0.30	0.36	− 0.03	− 0.42	1.30*	0.35	− 1.47	144.45	3.62	− 3.56	− 0.07	0.57	0.61	− 1.11*
P_3_ × P_5_	9.89	− 0.74	− 0.67	− 0.57	− 0.08	− 0.68	− 0.73	1.43	243.52*	38.01	10.57	− 0.39	17.30	0.90	1.96**
P_3_ × P_6_	10.33	0.04	− 0.09	0.12	− 0.39	− 0.06	− 0.29	− 0.62	− 134.86	− 23.88	2.12	− 1.95	16.53	− 0.84	− 0.44
P_3_ × P_7_	8.46	− 1.49	− 1.73	− 0.17	− 0.03	− 0.35	− 0.90	− 3.36	− 104.58	10.01	3.98	0.57	− 19.18	− 0.99	1.07*
P_3_ × P_8_	0.85	0.51	− 0.14	− 0.46	− 0.28	1.29*	0.41	0.18	110.86	− 14.92	4.21	− 2.64	20.52*	− 0.89	− 2.37**
P_3_ × P_9_	− 17.46	− 3.08*	− 2.54	− 0.07	0.39	− 2.86**	0.89	3.12	− 571.94**	− 48.94	− 3.41	− 4.30	− 17.98	0.43	− 1.36*
P_4_ × P_5_	− 3.27	0.96	0.46	0.68	0.02	0.22	− 1.93	− 0.32	282.07**	− 6.40	− 4.12	0.84	13.29	0.70	− 0.39
P_4_ × P_6_	− 1.54	2.98**	3.00**	0.22	− 0.45	0.95	− 3.32*	− 5.53*	406.76**	61.71*	20.56	2.43	52.16**	− 0.37	1.11*
P_4_ × P_7_	− 7.90	− 1.87	− 2.03	0.07	0.73*	− 0.15	0.41	2.73	− 210.35*	− 67.69**	− 10.88	− 2.48	− 49.49**	− 3.01**	− 2.64**
P_4_ × P_8_	4.64	2.05	1.73	0.07	0.57	0.67	2.88*	0.94	− 85.37	17.33	6.81	.00	− 18.46	1.96**	− 0.52
P_4_ × P_9_	− 8.17	− 0.62	− 0.16	− 0.65	0.50	− 2.71**	0.63	2.56	− 105.89	45.89	1.89	2.57	− 28.33*	− 0.60	0.91
P_5_ × P_6_	13.69	− 1.55	− 1.31	− 0.35	− 0.30	− 0.59	− 1.23	− 2.47	− 334.72**	− 24.91	− 1.07	− 0.44	− 24.03*	0.52	− 0.84
P_5_ × P_7_	0.46	− 0.38	− 0.58	0.36**	.00	− 0.22	− 1.17	− 1.71	18.98	24.01	− 14.12	− 2.51	26.95**	3.90**	1.50**
P_5_ × P_8_	24.44*	5.40**	5.53**	2.88	0.73*	0.15	0.97	− 2.33	403.43**	98.91**	27.97*	7.47**	11.43	− 3.28**	− 2.37**
P_5_ × P_9_	− 22.66	− 2.01	− 1.80	− 0.39	0.12	− 0.30	− 1.93	− 0.43	− 243.69	− 32.82	− 0.65	1.43	1.82	− 2.31*	1.79**
P_6_ × P_7_	− 6.57	0.45	0.68	0.67	1.01**	0.24	1.27	0.91	− 102.99	− 18.25	− 10.45	− 0.41	− 2.13	0.39	0.76
P_6_ × P_8_	− 4.22	0.36	0.12	− 1.31*	− 0.01	− 0.69	2.07	2.62	181.83	− 9.49	− 12.09	− 1.70	22.96*	1.42*	0.61
P_6_ × P_9_	− 15.84	0.08	− 0.04	0.85	0.68	0.61	0.72	7.44*	119.67	18.80	8.46	3.39	7.00	− 2.39*	− 0.39
P_7_ × P_8_	2.72	− 1.51	− 1.41	− 1.04	− 0.40	− 1.13*	− 1.87	− 1.45	− 108.55	− 4.56	− 3.40	− 1.32	− 26.78**	− 2.61**	− 2.38**
P_7_ × P_9_	− 2.56	3.62*	3.98*	1.32	0.18	0.62	− 0.27	3.38	302.83*	57.22	36.25*	4.57	63.49**	− 2.19*	0.47
P_8_ × P_9_	− 27.00	− 2.32	− 1.55	1.28	0.08	0.89	− 1.19	1.18	− 58.46	− 11.56	7.59	2.02	− 17.84	2.17	0.82
LSD (sij-slk)5%	18.70	2.06	2.05	1.09	0.71	1.11	2.59	4.24	189.23	48.7	21.83	3.11	18.75	1.40	0.89
LSD (sij-slk)1%	24.68	2.72	2.70	1.44	0.93	1.47	3.42	5.60	249.71	64.2	28.81	4.10	24.74	1.84	1.18
LSD (sij-skl)5%	16.55	1.82	1.81	0.96	0.62	0.98	2.29	3.75	167.40	43.1	19.31	2.75	16.58	1.24	0.79
LSD (sij-skl)1%	21.84	2.40	2.39	1.27	0.82	1.30	3.02	4.95	220.91	56.8	25.48	3.63	21.88	1.63	1.04

*LSD* least significant difference, *PH* plant height, *BD* base diameter, *CD* core diameter, *MD* middle diameter, *TD* top diameter, *NN* number of nodes, *DTFF* days to 1st flowering, *D50%F* days to 50% flowering, *FW1* fresh stem weight with leaves and pod, *FW2* fresh stem weight without leaves and pod, *DSW* dry stick weight, *DFW* dry fiber weight, *NF* number of pods per plant, *NS* number of seeds per pod, *SW* 1000 seeds weight. *Significant at P ≤ 0.05 level, **Highly significant at P ≤ 0.01 level.

### Estimation of heterosis effect of kenaf hybrids for yield and yield components and morphological traits

Heterosis was calculated using the relative performance of hybrids with mid-parent (MPH) and better-parent (BPH) values. Parent heterosis is favored over mid-parent heterosis in hybrid formation. High positive heterosis values are preferable for yield production traits, whereas negative values are favored for node number and 1000 seed weight.

### Relative heterosis response in pooled environments

Percentage heterosis relative to mid-parents (MPs) was significantly positive in 13 out of 36 crosses for dry fiber weight (25.74–91.94), five crosses for dry stick weight (49.3–71.65), and 20 crosses for number of pods per plant (49.22–108.72). However, mid-parents of 18 crosses for fresh stem weight with leaves and pod (32.75–91.43) and five crosses for fresh stem weights without leaves and pod (46.16–56.76) were significantly positive (Table 9). For 1000 seed weight, 29 crosses out of 36 had negative heterosis (in the desired direction). This indicates that they had smaller-sized seeds. Over dominance was observed in cross P_5_ × P_8_ for base diameter, core diameter, middle diameter, fresh stem weight with leaves and pods, and fresh stem weight without leaves and pods, as well as cross P_4_ × P_6_ for base diameter, core diameter, fresh stem weight with leaves and pods, fresh stem weight without leaves and pods, and pods number per plant and the cross P_2_ × P_3_ for base diameter and core diameter, with a high potency’s ratio. Furthermore, the crosses P_2_ × P_3_ and P_2_ × P_5_ for fiber yield, and the crosses P_1_ × P_4_ and P_4_ × P_9_ for stick yield features had the highest mid parent heterosis. Due to the existence of over dominance, crosses P_1_ × P_4_, P_2_ × P_8_ and P_7_ × P_9_ were chosen for pods number per plant for seed yield. Negative heterosis estimates for the nodes number and 1000 seed weight are desirable, where small values for these traits indicate good fiber quality and smaller seed size, therefore, negative magnitude implies high heterosis. Cross P_2_ × P_9_ had the lowest negative nodes number value (− 11.02) for quality fiber yield, and cross P_1_ × P_5_ with the lowest negative heterosis for 1000 seed weight (− 18.76) would produce well where smaller seed size kenaf accessions perform well.

### Heterobeltiosis response exposed to a pooled environment

Overdominance for heterobeltiosis was seen in the most outstanding crosses P_2_ × P_3_ and P_2_ × P_5_ for fiber yield and the crosses P_1_ × P_4_ and P_4_ × P_9_ for stick yield, as shown in Table 9, since their heterobeltiosis values were very significant positive with high potence ratio. Due to the presence of over dominance, the seed yield crosses P_1_ × P_4_, P_1_ × P_8_ and P_7_ × P_9_ were chosen for pods number per plant. Overdominance for heterobeltiosis, as indicated by the potence ratio, was found in the promising crosses P_2_ × P_3_, P_5_ × P_8_ and P_7_ × P_9_ for base diameter and core diameter, cross P_6_ × P_7_ for top diameter, cross P_5_ × P_8_ for middle diameter, fresh stem weight with leaves and pods, and fresh stem weight without leaves and pods, and cross P_4_ × P_6_ for base diameter, core diameter, fresh stem weight with leaves and pods, and pods number per plant. In terms of node number and seed weight, negative heterosis is preferable. Meanwhile, in terms of percentage F_1_ heterosis above high parent in node number and 1000 seed weight, crosses P_2_ × P_9_ and P_1_ × P_5_ had the lowest negative heterosis values, respectively.

**Table 9:** Estimates of mid-parent and better parent heterosis for 15 characteristics of 36 crosses in kenaf over two environments.

Hybrids	PH		BD		CD		MD		TD		NN		DTFF		D50%F	
	MPH	BPH	MPH	BPH	MPH	BPH	MPH	BPH	MPH	BPH	MPH	BPH	MPH	BPH	MPH	BPH
P_1_ × P_2_	5.71	2.08	11.39	2.09	12.70	2.66	2.67	− 7.80	− 16.57	− 18.04	29.70	3.74	− 0.78	1.28	− 7.83	-13.49
P_1_ × P_3_	3.93	− 1.61	15.87*	13.51*	18.12	16.30	2.62	1.32	16.36	12.90	57.74**	33.59	.00	− 3.53	1.13	-1.37
P_1_ × P_4_	0.09	− 5.82	13.51	12.91	14.96	14.66	4.37	3.87	− 5.79	− 5.86	41.09*	22.63	− 4.46	− 5.06	− 7.63	-9.92
P_1_ × P_5_	− 8.70	− 11.80	− 0.87	− 3.05	0.11	− 1.68	− 12.34	− 12.56	− 3.33	− 3.83	21.95	18.12	6.43	3.53	3.65	1.37
P_1_ × P_6_	1.71	0.16	9.26	− 1.06	13.44	1.40	0.69	− 4.35	6.47	4.48	42.14*	23.46	− 0.16	− 0.32	− 2.14	-4.69
P_1_ × P_7_	8.52	2.54	15.98	8.35	20.75*	13.24*	0.49	− 4.96	− 18.07	− 20.06	30.53	23.46	3.60	− 3.21	− 2.29	-6.32
P_1_ × P_8_	6.14	3.92	11.80	2.36	13.22	3.79	1.81	− 0.70	− 11.63	− 11.79	13.28	12.54	− 0.96	− 0.96	− 2.89	-3.02
P_1_ × P_9_	− 2.94	− 7.25	− 4.42	− 8.90	− 4.49	− 9.40	− 7.28	− 12.78	1.94	− 4.45	− 7.82	− 8.20	5.75	3.21	7.87	3.57
P_2_ × P_3_	− 3.61	− 11.70	37.32**	28.28**	45.81**	34.73**	13.45	0.73	10.74	5.60	109.27**	95.10**	5.37	− 0.31	− 3.29	-11.33
P_2_ × P_4_	8.72	− 0.99	20.25*	10.75*	23.12*	11.89*	14.40	2.30	− 6.62	− 8.34	85.24**	67.85**	3.59	2.15	− 3.51	-7.23
P_2_ × P_5_	1.82	− 4.90	16.75	9.24	22.09*	13.08*	3.51	− 7.26	− 21.71	− 22.70	55.81**	21.69	9.03	4.00	4.59	-3.86
P_2_ × P_6_	7.15	1.95	21.85*	20.26*	26.30*	23.68*	8.19	1.95	− 12.38	− 15.50	73.40**	57.25*	− 3.14	− 5.23	− 11.39	-14.70
P_2_ × P_7_	6.17	− 2.92	18.10	15.69	19.18	15.53	7.98	2.19	− 14.09	− 17.63	33.42	11.58	− 6.71	− 14.46	− 16.15	-24.34
P_2_ × P_8_	6.38	0.66	14.29	14.16	15.93	15.13	8.32	− 0.49	− 0.16	− 1.76	36.18	9.47	− 5.81	− 7.69	− 8.48	-14.22
P_2_ × P_9_	− 12.51	− 19.13	− 10.90	− 14.50	− 10.05	− 13.82	− 4.58	− 9.21	3.38	− 4.69	− 11.02	− 28.61	1.61	− 2.77	− 4.27	-13.49
P_3_ × P_4_	4.71	4.71	16.61*	16.61*	18.26	18.26	5.67	5.67	− 0.97	− 0.97	75.25**	75.25**	0.33	0.33	− 5.08	-5.08
P_3_ × P_5_	5.46	3.27	5.92	5.74	7.50	7.22	− 0.65	− 1.67	0.77	− 2.72	14.87	− 5.22	− 0.17	− 1.02	2.88	2.59
P_3_ × P_6_	6.93	2.74	14.04	5.24	16.07	5.20	4.40	− 2.03	− 1.29	− 2.44	46.34*	42.03	− 1.50	− 4.82	− 3.01	-7.81
P_3_ × P_7_	7.34	7.11	4.41	− 0.54	3.51	− 1.50	4.01	− 2.80	6.84	6.23	19.76	6.47	− 3.74	− 6.90	− 10.00	-11.56
P_3_ × P_8_	4.75	1.22	17.19	9.36	15.64	7.54	2.32	− 1.44	0.74	− 2.43	52.65**	29.98	1.00	− 2.56	− 1.55	-3.86
P_3_ × P_9_	− 1.26	− 2.22	0.42	− 2.36	1.75	− 2.03	− 2.04	− 8.94	17.55	13.44	10.73	− 5.91	3.58	2.36	2.79	1.16
P_4_ × P_5_	− 0.03	− 2.73	16.46*	14.50*	16.41	14.03	13.20	12.94	3.75	3.13	28.09	8.37	− 4.42	− 7.59	− 2.60	-7.05
P_4_ × P_6_	1.81	− 2.79	30.64**	18.88**	35.71**	21.02**	9.21	3.26	− 1.94	− 3.69	63.65**	63.50*	− 9.09	− 9.81	− 12.91	-13.02
P_4_ × P_7_	0.81	0.36	6.80	0.27	5.90	− 0.93	9.99	3.56	25.34	22.38	21.89	11.42	− 2.90	− 9.81	− 1.81	-8.09
P_4_ × P_8_	5.49	1.28	27.60**	17.40**	28.69**	17.70**	10.59	7.36	21.08	20.78	40.04*	22.40	3.82	3.16	− 2.68	-5.22
P_4_ × P_9_	0.24	− 1.38	22.40**	17.26**	24.89*	18.18*	4.76	− 1.89	21.65	14.12	10.29	− 3.79	− 2.77	− 5.70	− 5.85	-11.75
P_5_ × P_6_	6.96	4.90	3.97	− 3.91	7.05	− 2.75	2.24	− 3.12	− 4.66	− 6.92	14.03	− 3.59	− 3.63	− 6.11	− 5.46	-9.90
P_5_ × P_7_	3.19	0.85	6.14	1.28	6.74	1.83	10.42	4.19	1.55	− 1.42	4.82	− 3.80	− 4.59	− 8.47	− 6.45	-8.33
P_5_ × P_8_	12.46	10.92	35.43**	26.59**	42.06**	32.43**	32.25**	28.67**	18.55	18.14	14.58	10.28	1.81	− 0.96	− 5.20	-7.16
P_5_ × P_9_	− 7.34	− 8.38	− 3.91	− 6.41	− 2.16	− 5.56	0.68	− 5.50	− 7.32	− 13.54	7.23	3.45	− 3.04	− 3.37	− 1.61	-3.45
P_6_ × P_7_	1.82	− 2.37	14.96	11.18	19.42	13.43	13.07	12.56	31.71	30.93	27.51	16.46	− 1.72	− 8.04	− 4.18	-10.42
P_6_ × P_8_	2.79	2.19	19.25*	17.82*	21.13	17.80	− 3.95	− 6.52	6.92	4.75	15.89	1.22	1.77	1.60	0.67	-2.08
P_6_ × P_9_	− 1.25	− 4.21	18.21*	12.01*	22.15*	14.69*	9.64	8.53	23.82	18.16	62.02**	41.22*	− 4.28	− 6.43	4.31	-2.34
P_7 ×_ P_8_	6.67	2.86	7.18	4.88	7.94	5.35	0.59	− 2.53	− 3.01	− 5.53	− 6.20	− 10.73	− 7.03	− 13.14	− 8.18	-11.85
P_7_ × P_9_	7.78	6.51	24.86**	22.25**	30.62**	29.04**	20.77*	20.08	8.65	4.28	14.82	9.02	− 9.15	− 13.13	− 8.52	-8.66
P_8_ × P_9_	− 1.20	− 3.62	11.24	6.64	14.33	10.27	22.60*	18.13	6.39	− 0.44	32.91*	32.59	− 4.76	− 7.05	− 5.73	-9.37
Mean	2.69	− 0.93	13.61	8.47	15.97	10.07	5.95	1.13	2.91	0.13	32.84	19.56	− 1.03	− 3.80	− 3.69	-7.40
LSD5%	35.42	40.90	3.90	4.50	3.88	4.48	2.06	2.38	1.34	1.55	2.11	2.43	4.90	5.66	8.03	9.28
LSD1%	46.75	53.98	5.14	5.94	5.12	5.91	2.72	3.14	1.77	2.04	2.78	3.21	6.47	7.47	10.60	12.24

Hybrids	FW1		FW2		DSW		DFW		NF		NS		SW			
	MPH	BPH	MPH	BPH	MPH	BPH	MPH	BPH	MPH	BPH	MPH	BPH	MPH	BPH		
P_1_ × P_2_	35.12	7.74	18.62	− 0.11	16.17	4.26	17.29	− 2.81	67.52*	32.78	− 34.66	− 42.18	− 0.24	− 0.82		
P_1_ × P_3_	46.15*	35.44*	14.88	14.35	12.71	− 0.76	27.35*	18.06	73.68**	57.68*	− 1.60	− 2.65	− 0.79	− 4.07		
P_1_ × P_4_	32.75*	32.21*	20.74	16.87	65.88**	63.08*	21.72	17.32	103.33**	80.76**	− 0.79	− 0.91	3.16	− 2.39		
P_1_ × P_5_	14.40	− 1.28	− 5.58	− 17.20	19.01	10.01	− 8.60	− 20.96	6.77	3.31	− 17.7	− 21.53	− 18.76	− 23.34		
P_1_ × P_6_	32.48	20.85	14.68	− 3.88	30.87	15.22	13.24	− 5.98	33.57	26.24	− 22.69	− 23.21	− 9.68	− 10.19		
P_1_ × P_7_	43.72*	31.64*	19.40	6.67	17.52	19.08	27.50	2.65	55.99**	33.54	− 11.34	− 20.68	− 4.73	− 6.87		
P_1_ × P_8_	18.82	7.44	4.99	− 1.66	15.57	14.79	8.34	2.01	77.09**	74.07**	− 3.50	− 5.75	− 2.84	− 4.46		
P_1_ × P_9_	− 6.28	− 7.31	6.90	− 4.97	− 3.71	− 10.03	7.32	− 7.83	49.27*	35.33	− 8.91	− 11.71	− 6.59	− 8.93		
P_2_ × P_3_	86.42**	58.17**	48.83**	24.85	11.22	− 10.74	91.94**	69.82**	24.74	7.12	− 29.6	− 38.27	0.19	− 2.57		
P_2_ × P_4_	37.54	10.02	33.97	9.87	46.66	33.66	48.60**	26.93	85.00**	62.09*	− 20.11	− 29.22	0.93	− 3.96		
P_2_ × P_5_	60.27*	45.81*	36.20	29.89	35.15	30.87	56.74**	48.97**	41.54	9.51	− 17.10	− 29.61	− 1.51	− 6.54		
P_2_ × P_6_	34.01	15.35	48.26*	47.41	24.17	21.50	47.93**	47.55*	30.20	− 0.98	8.95	− 3.01	− 5.27	− 5.29		
P_2_ × P_7_	61.86**	38.79**	31.51	22.93	26.19	11.96	53.63**	48.13*	52.07*	7.72	0.27	− 0.96	− 7.12	− 8.67		
P_2_ × P_8_	51.50*	31.48*	18.33	5.51	12.84	0.67	25.93	9.83	108.24**	67.21**	− 33.66	− 42.48	4.92	3.76		
P_2_ × P_9_	− 34.08	− 46.99	− 4.66	− 10.41	1.03	− 3.25	3.12	− 1.22	6.48	− 21.42	− 25.59	− 35.91	1.64	− 0.34		
P_3_ × P_4_	51.33**	51.33**	21.51	21.51	24.69	24.69	25.74*	25.74*	42.51	42.51	− 2.54	− 2.54	− 5.79	− 5.79		
P_3_ × P_5_	66.14**	53.72**	32.33	15.59	28.18	5.48	22.39	13.47	51.15*	33.23	− 4.12	− 7.64	1.18	− 1.34		
P_3_ × P_6_	16.52	14.53	10.24	− 7.95	17.72	− 7.06	12.98	0.18	61.63**	39.50	− 7.67	− 9.26	− 5.69	− 8.31		
P_3_ × P_7_	19.70	18.19	19.53	6.35	15.07	2.48	27.25	9.07	9.11	− 13.75	− 11.29	− 21.38	− 1.83	− 2.93		
P_3_ × P_8_	50.95*	46.97*	10.90	3.43	19.10	5.49	6.79	5.05	73.74**	60.23*	− 12.29	− 13.43	− 12.69	− 14.17		
P_3_ × P_9_	− 13.98	− 19.45	− 1.33	− 12.64	2.88	− 14.55	6.20	− 2.29	19.46	− 0.65	− 1.83	− 3.84	− 7.11	− 7.90		
P_4_ × P_5_	71.51**	48.51**	22.62	4.58	37.23	28.88	29.91*	16.03	49.97*	29.55	− 2.58	− 7.22	− 5.57	− 5.84		
P_4_ × P_6_	74.52**	59.79**	46.16**	19.33	66.04*	48.39	36.11*	16.51	108.72**	76.64*	− 3.47	− 3.99	− 0.12	− 4.99		
P_4_ × P_7_	14.24	5.03	− 0.52	− 13.63	20.85	17.30	14.27	− 5.29	− 20.37	− 38.12	− 17.34	− 25.96	− 12.74	− 15.61		
P_4_ × P_8_	34.10	21.70	26.45	14.90	44.43	41.06	20.11	17.22	26.79	14.46	0.65	− 1.82	− 6.26	− 9.85		
P_4_ × P_9_	46.56**	45.53**	48.19**	28.04	71.65**	62.98*	46.13**	29.58*	15.61	− 5.62	1.48	− 1.76	2.63	− 0.48		
P_5_ × P_6_	− 2.61	− 8.44	13.46	7.61	26.37	19.82	20.06	14.38	9.74	7.10	− 2.84	− 7.96	− 9.00	− 13.66		
P_5_ × P_7_	37.76	28.97	29.99	27.28	4.88	− 4.20	10.25	1.22	49.22**	31.42	8.53	− 6.88	− 2.44	− 5.91		
P_5_ × P_8_	91.43**	81.65**	56.76**	46.05*	57.78*	44.94	52.34**	39.1*	51.94*	44.60	− 20.8	− 22.73	− 14.93	− 18.41		
P_5_ × P_9_	25.67	9.47	9.99	8.29	28.87	27.39	30.04	28.98	30.97	22.42	− 12.74	− 14.21	− 3.09	− 6.29		
P_6_ × P_7_	16.64	16.11	12.57	4.66	6.06	− 7.66	19.94	15.36	31.50	18.35	− 2.28	− 12.05	− 4.06	− 5.68		
P_6_ × P_8_	55.95**	54.44**	15.71	2.65	9.03	− 4.56	7.49	− 6.05	75.20**	62.92**	− 1.00	− 3.94	− 4.78	− 5.84		
P_6_ × P_9_	44.04*	32.74*	27.75	19.40	31.04	22.90	33.13*	27.83	67.02**	59.80**	− 3.99	− 7.54	− 8.05	− 9.85		
P_7 ×_ P_8_	24.35	22.59	14.78	9.09	14.26	13.54	10.58	− 6.51	10.77	− 6.52	− 19.99	− 29.89	− 14.65	− 15.15		
P_7_ × P_9_	62.00**	49.91**	35.35	34.59	49.30*	37.83	44.14*	33.33	89.89**	78.09**	− 9.79	− 21.48	− 7.66	− 7.91		
P_8_ × P_9_	49.18**	36.25**	9.29	3.32	31.03	21.66	17.07	6.10	49.43*	33.40	− 1.11	− 1.88	− 7.39	− 8.18		
Mean	37.52	26.36	21.36	10.63	26.05	16.31	25.97	14.76	47.76	29.57	− 9.58	− 14.87	− 4.91	− 7.19		
LSD5%	358.38	413.82	92.18	106.44	41.34	47.74	5.89	6.80	35.50	40.99	2.65	3.06	1.69	1.95		
LSD1%	472.94	546.10	121.64	140.46	54.56	63.00	7.77	8.97	46.85	54.10	3.49	4.04	2.23	2.57		

*PH* plant height, *BD* base diameter, *CD* core diameter, *MD* middle diameter, *TD* top diameter, *NN* number of nodes, *DTFF* days to 1st flowering, *D50%F* days to 50% flowering, *LSD* least significant difference, *FW1* fresh stem weight with leaves and pod, *FW2* fresh stem weight without leaves and pod, *DSW* dry stick weight, *DFW* dry fiber weight, *NF* number of pods per plant, *NS* number of seeds per pod, *SW* 1000 seeds weight. **Highly significant at P ≤ 0.01 level, *Significant at P ≤ 0.05 level.

### Selection of best parents and offspring

The yield and yield contributing components of fifteen parents and crosses were investigated in this study. Table 10 lists the best general and specific combiners based on trait performance. It's always possible that some crosses will work better in some characters than others. To determine the most favorable, rate each on a character-by-character basis and see which ones perform better across the range. When the SCA results of 36 crosses were reviewed, the cross P_5_ × P_8_ was shown to be the best in ranking for all the characters. P_7_ × P_9_, P_1_ × P_3_, P_2_ × P_3_, P_1_ × P_7_, P_4_ × P_8_, and P_2_ × P_4_ were the next crosses (Table 11). This was possibly due to their greater distance from the others. The performance of the cross P_5_ × P_9_ was determined to be extremely low. This was because these two genotypes are the most similar. However, based on their cumulative rating, ten superior crossings were selected for inclusion in a future breeding effort. Thirty-six kenaf F_1_s were arrayed in order of merit, with the rank total against the crosses indicating the lot's position.

**Table 10:** Best general and specific combiner for 15 traits.

Sl No.	Traits	General combiner	Specific combiner
1	Plant height (cm)	P_7_ (BJRI Kenaf4)	P_5_ (ML36-25) × P_8_ (MLRing4P2)
2	Base diameter (mm)	P_4_ (ML36-24)	P_2_ (ML9) × P_3_ (ML36-10)
3	Core diameter (mm)	P_4_ (ML36-24)	P_2_ (ML9) × P_3_ (ML36-10)
4	Middle diameter (mm)	P_4_ (ML36-24)	P_5_ (ML36-25) × P_7_ (BJRI Kenaf4)
5	Top diameter (mm)	P_4_ (ML36-24)	P_6_ (ML36-27) × P_7_ (BJRI Kenaf4)
6	Number of nodes	P_1_ (ML5)	P_2_ (ML9) × P_9_ [ML36-21(2)]
7	Days to first flowering	P_2_ (ML9)	P_2_ (ML9) × P_5_ (ML36-25)
8	Days to 50% flowering	P_2_ (ML9)	P_6_ (ML36-27) × P_9_ [ML36-21(2)]
9	Fresh stem weight with leaves and pod (g)	P_4_ (ML36-24)	P_2_ (ML9) × P_3_ (ML36-10)
10	Fresh stem weight without leaves and pod (g)	P_4_ (ML36-24)	P_5_ (ML36-25) × P_8_ (MLRing4P2)
11	Dry stick weight (g)	P_4_ (ML36-24)	P_7_ (BJRI Kenaf4) × P_9_ [ML36-21(2)]
12	Dry fiber weight (g)	P_4_ (ML36-24)	P_2_ (ML9) × P_3_ (ML36-10)
13	Number of pods per plant	P_7_ (BJRI Kenaf4)	P_7_ (BJRI Kenaf4) × P_9_ [ML36-21(2)]
14	Number of seeds per pod	P_9_ [ML36-21(2)]	P_2_ (ML9) × P_6_ (ML36-27)
15	1000 seeds weight (g)	P_2_ (ML9)	P_1_ (ML5) × P_5_ (ML36-25)

**Table 11:** Rank positions of the F_1’_s based on 15 morpho-physiological characters.

Crosses	PH	BD	CD	MD	TD	NN	DTFF	D50%F	FW1	FW2	DSW	DFW	NF	NS	SW	Cumulative rank	Position of crosses
P_1_ × P_2_	6	17	19	15	27	11	29	31	16	19	21	28	17	34	22	312	26
P_1_ × P_3_	14	7	6	10	7	33	24	17	9	17	19	6	7	4	28	208	3
P_1_ × P_4_	23	15	14	14	26	22	33	33	18	15	2	16	3	8	35	277	18
P_1_ × P_5_	34	30	30	36	17	21	3	10	29	34	26	35	34	26	1	366	34
P_1_ × P_6_	20	22	17	17	12	26	18	21	14	20	8	19	25	36	6	281	20
P_1_ × P_7_	3	5	5	26	36	30	4	16	8	8	15	7	10	18	20	211	5
P_1_ × P_8_	10	19	18	24	34	10	22	24	25	28	28	22	11	5	29	309	25
P_1_ × P_9_	29	31	33	9	5	4	8	2	33	9	36	14	29	12	14	268	15
P_2_ × P_3_	30	1	1	4	9	36	7	19	1	2	23	1	23	33	19	209	4
P_2_ × P_4_	2	12	12	8	23	34	5	12	21	16	10	11	8	25	18	217	7
P_2_ × P_5_	17	8	8	27	35	35	1	4	12	12	7	4	19	20	21	230	9
P_2_ × P_6_	5	9	9	12	32	29	25	34	20	6	17	12	24	1	10	245	12
P_2_ × P_7_	11	10	10	21	33	20	34	36	7	13	6	9	14	3	7	234	10
P_2_ × P_8_	12	25	26	23	15	17	35	28	17	26	30	26	4	35	36	355	32
P_2_ × P_9_	36	36	36	33	1	1	17	3	36	36	31	36	35	17	12	366	35
P_3_ × P_4_	13	18	15	20	30	32	16	26	11	21	27	21	21	13	9	293	21
P_3_ × P_5_	8	26	25	31	22	7	21	11	6	7	5	23	12	10	34	248	13
P_3_ × P_6_	7	21	21	16	28	15	20	23	30	30	16	30	13	22	15	307	24
P_3_ × P_7_	9	27	31	25	21	9	23	32	26	18	14	18	30	24	30	337	29
P_3_ × P_8_	18	13	22	30	24	31	14	18	15	27	13	33	9	23	4	294	22
P_3_ × P_9_	32	35	35	22	11	2	11	6	35	33	25	34	27	15	8	331	28
P_4_ × P_5_	24	11	13	6	18	18	31	20	5	23	29	17	15	11	16	257	14
P_4_ × P_6_	21	4	4	13	31	28	36	35	2	3	4	10	2	19	31	243	11
P_4_ × P_7_	27	32	34	18	3	14	15	7	31	35	33	31	36	31	2	349	31
P_4_ × P_8_	15	6	7	19	8	25	2	14	23	14	12	20	28	7	13	213	6
P_4_ × P_9_	28	24	23	32	10	3	13	9	27	5	18	8	33	21	27	281	19
P_5_ × P_6_	4	29	27	28	25	8	28	30	34	31	22	25	31	14	11	347	30
P_5_ × P_7_	19	23	24	11	19	13	26	27	19	10	35	32	5	2	32	297	23
P_5_ × P_8_	1	2	2	1	4	16	10	29	3	1	3	2	16	32	5	127	1
P_5_ × P_9_	33	33	32	29	14	12	32	22	32	32	20	15	20	28	33	387	36
P_6_ × P_7_	26	14	11	7	2	19	9	15	24	29	32	24	22	16	25	275	17
P_6_ × P_8_	25	16	16	35	20	6	6	8	10	24	34	29	6	9	24	268	16
P_6_ × P_9_	31	20	20	5	6	23	12	1	13	11	9	5	18	29	17	220	8
P_7 ×_ P_8_	16	28	28	34	29	5	30	25	28	22	24	27	32	30	3	361	33
P_7_ × P_9_	22	3	3	2	13	24	19	5	4	4	1	3	1	27	23	154	2
P_8_ × P_9_	35	34	29	3	16	27	27	13	22	25	11	13	26	6	26	313	27

The lowest value for each character indicates the best one, *PH* plant height, *BD* base diameter, *CD* core diameter, *MD* middle diameter, *TD* top diameter, *NN* number of nodes, *DTFF* days to 1st flowering, *D50%F* days to 50% flowering, *FW1* fresh stem weight with leaves and pod, *FW2* fresh stem weight without leaves and pod, *DSW* dry stick weight, *DFW* dry fiber weight, *NF* number of pods per plant, *NS* number of seeds per pod, *SW* 1000 seeds weight.

## Discussion

The wide variation in the crop's botanical and agro morphological properties suggests that the genotypes are genetically diverse (Ogunniyan, 2016). Segregation was seen in 36 crosses for stem color, leaf shape, leaf color (lamina), petiole color, pod shape, seed shape, and seed coat color in the F_1_ generation (Supplementary Fig. 1). In addition, some of the crosses indicated significant differences in leaf shape and pigmentation pattern on the stem and petiole (Table 3). Indeed, the green morphotype kenaf leaves are more valuable for their food value, and their fibers are better for stringing^22^. The parents had green, green with reddish patches, and purple stem color, however the cross combinations had green, green with reddish patches, purple, reddish above greenish below, reddish, and red stem color. When the upper surface light reddish but lower surface green pigmented parent P_7_ was crossed with the rest of the green parents, the F_1_ showed various pigmentation patterns on the petiole, including green, upper surface light reddish but lower surface green, reddish, and purple. When the ash gray, brownish, and black with few brownish seed coat color parents crossed, F_1_ offspring with blackish, ash gray, brownish, and black with few brownish seeds were produced, as were those observed by Osman et al. (2011). In addition, the leaf shape, leaf color (lamina), pod shape, and seed shape of selected parents and their F_1_ hybrids showed a wide range of diversity.

Table 4 shows the results of the mean squares analysis of variance on the combined data of the two environments, which revealed that, except for middle diameter, the effects of the environments were highly significant (p ≤ 0.01). The genotypes performed differently across the environments studied, based on the analysis results on the combined data. The effects of genotype by environment interaction were highly significant (p ≤ 0.01) for all traits measured except for plant height, and significant (p ≤ 0.05) for days to first flowering. These results indicate that the effects of genes controlling these traits were expressed differently at different environments. The mean squares of GCA were bigger than the mean squares of SCA for all traits except top diameter, nodes number, and fresh stem weight with leaves and pods in our investigation indicating that the parental materials studied had a lot of genetic variability. Due to the presence of large additive gene effects, this result usually favors the selection strategy of breeding^39,40^. Days to first flowering, dry fiber weight and 1000-seed weight were found to indicate the prevalence of additive gene effects in the development of the features by Mostofa et al.^29^. For seed yield per plant and number of pods per plant in kenaf, Mukewar et al.^31^ found that additive gene action predominated. For yield components such as plant height, fresh and dry weight of bark, and usable stick in kenaf, Pace et al.^32^ found additive gene action was more relevant. All the analyzed variables (except top diameter and node number) had large mean square GCA values, indicating that the parental materials studied had a lot of genetic variability. All the examined parameters (excluding plant height, days to 50% flowering, fresh stem weight without leaves and pod) showed a very significant effect on GCA environment interaction, indicating that environmental variation influenced additive gene action. Furthermore, environments had a significant influence on how these characters changed^1^. Using combining ability investigations, Mostofa et al.^30^ and Youcai et al.^49^ found similar results in *Hibiscus cannabinus*, Sobhan^43^ in *Hibiscus sabdariffa*, and Khatun^23^ in *Corchorus capsularis*.

Table 4 shows that except for plant height and seeds number per pod, genotypes and environment interaction were highly significant in the pooled quantitative data showed that the environment significantly impacted both parents and offspring for all attributes. We employed nine kenaf genotypes with a range of morpho-physiological and yield features. The mean squares of GCA were bigger than the mean squares of SCA for all traits except top diameter, nodes number, and fresh stem weight with leaves and pods in our investigation indicating that the parental materials studied had a lot of genetic variability. Due to the presence of large additive gene effects, this result usually favors the selection strategy of breeding^39,40^. Days to first flowering, fibre weight per plant, and 1000-seed weight were found to indicate the prevalence of additive gene effects in the development of the features by Mostofa et al.^29^. Days to first flowering and plant height were controlled by one dominant gene pair, while raw fibre yield was controlled by three (Thombre and Patil, 1985). For seed yield per plant and number of pods per plant in kenaf, Mukewar et al.^31^ found that additive gene action predominated. For yield components such as plant height, fresh and dry weight of bark, and usable stick in kenaf, Pace et al.^32^ found additive gene action was more relevant. When both general and specific combining ability effects are essential, Comstock et al. (1949) suggested using a reciprocal recurrent selection strategy to generate high yielding varieties. All the examined parameters (excluding plant height, days to 50% flowering, fresh stem weight without leaves and pod) showed a very significant effect on GCA environments, indicating that environmental variation influenced additive gene action. Furthermore, environments had a big influence on how these characters changed^1^.

The findings revealed that environmental influences controlled the expression of kenaf phenotypic features. The mean of hybrids is higher than the parental mean for plant height, base diameter, core diameter, middle diameter, top diameter, number of nodes, fresh stem weight with leaves and pod, fresh stem weight without leaves and pod, dry stick weight, dry fiber weight, and pods number per plant. In comparison to their parents, hybrids have higher fiber and stick yields and a higher seed yield. Days to first flowering and days to 50% flowering of hybrids, on the other hand, were lower than the parental mean. However, the mean hybrid of 1000 seeds weight had a lower mean value than the parental mean, indicating that smaller seed size hybrids were preferable to parents, but that when the nodes number was larger than the parental mean, the fiber production per plant was reduced. Parent P_1_ had the highest fiber weight per plant (26.71 g) among the parental lines, followed by P_4_ (24.78 g), and P_8_ (23.59 g). Similarly, the hybrids P_2_ × P_3_, P_4_ × P_6_, and P_5_ × P_8_ had much greater fiber yield (38.74 g, 28.87 g, and 32.81 g, respectively) than their respective parents. Parent P_3_ produced the meanest stick weight per plant (123.24 g), followed by P_7_ (96.27 g), and P_8_ (95.06 g). The hybrids P_1_ × P_4_, P_5_ × P_8_, and P_7_ × P_9_ generated the highest mean stick weight per plant (152.93 g, 137.78 g, and 132.68 g, respectively), which was significantly higher than both parents. The cross combinations P_1_ × P_4_, P_4_ × P_6_, and P_7_ × P_9_ produced the most pods per plant (157, 172 and 217, respectively), far exceeding any of the parents, demonstrating the presence of transgressive segregation in the cross.

The results of combining ability analysis are used to select parents and crosses. However, the nature of gene action revealed that both additive and non-additive gene effects were crucial in governing the diverse characters of kenaf in this investigation, but non-additive gene action was shown to control most of the characters. Gupta and Singh^16^ state that non-additive gene activity affects plant height, basal stem diameter, fiber weight per plant, and stick weight per plant (*H. sabdariffa*). Non-additive gene action predominated for all other parameters except days to first flowering, dry fiber weight, and 1000 seed weight. Through pedigree and single seed descent methods of breeding, it is possible to improve traits with a predominance of additive genetic effects. When non-additive gene effects predominated, however, bi-parental mating and a recurrent selective breeding system would be an effective way to obtain hybrid diversity.

Parents' breeding potential is usually linked to their GCA effects. Parents having high GCA effects for certain qualities could be employed as donor parents in hybridization programs to increase these features. A low or negative combining ability effect reflects a plant's inability to pass on its genetic superiority to hybrids^10^. Positive values with the highest magnitudes have the most impact. On the other hand, the greatest negative values have the least impact^44^. Additive genetic variance is a major contributor to the GCA component. As a result, each parent's GCA variation has a major impact on the parents' decisions. A good general combiner is a parent with a higher positive significant GCA effect^36^. In this study, GCA's high value for the traits of interest was distributed across genotypes, showing that none of the genotypes used had the optimal combination of GCA values for the several characters of interest.

Parent P_4_ (ML36-24) was the best parental line in terms of base diameter, core diameter, middle diameter, fresh stem weight with leaves and pod, fresh stem weight without leaves and pods, dry stick weight, and dry fiber weight content, showing the accumulation of favorable additive genes for these traits in the hybrid. The parent P_3_ had positive effects on plant height, fresh stem weight without leaves and pods, dry stick weight, and dry fiber weight, conversely the parent P_1_ was the good general combiner for days to first flowering and dry fiber weight, and it contributed positively to the hybrid for these traits. The major goal of this breeding program is to develop high-yielding hybrids with high potential fiber production as compared to existing cultivars or on par with them. As a result, for fiber yield per plant, parent P_4_ might be well combined. Furthermore, the parent P_3_ had positive effects on plant height, fresh stem weight without leaves and pods, dry stick weight, and dry fiber weight, and the parent P_1_ was the best general combiner for days to first flowering, dry fiber weight, and number of pods per plant. Other parents are specialized in one or two characteristics. To put it briefly, the parental lines P_1_, P_3_, and P_4_ were determined to be outstanding general combiners for fiber yield and yield-related parameters. Parents P_3_ and P_4_ were good general combiners in terms of stick yield. The parents P_1_, P_6_ and P_7_, on the other hand, were good general combiners for seed yield (pods number per plant). In contrast, parent P_2_ was chosen as a good general combiner for the first flowering and days to 50% flowering features, which are important for late maturity cultivars. Golam et al.^14^ found that 50% flowering and days to maturity, in addition to other morpho-agronomic features, may be the two most important variables in classifying kenaf accessions.

Parents with a high GCA for a certain trait and high adaptability indicate additive gene action. Because additive variance may be fixed, selecting for qualities regulated by additive variance is a very effective strategy^35^. The parents P_3_ and P_4_ were found to be the finest general combiners, with extremely significant values for fresh stem weight without leaves and pod, dry stick weight, and dry fiber weight, all of which are more suitable for desired kenaf features such as bast fiber and core fiber production. The parents with the highest GCA values (strong GCA effects) could be used to improve the kenaf population in Malaysia through varietal development based on desirable features.

In general, crosses with high × low general combiners for yield components outperform others. According to an investigation of combining ability impacts, high specific combiners involved high × high, high × low, high × average, average × average, average × low, and low × low combining parents. In crosses with high × low and low × low general combiners, Jinks^21^ described severe SCA effects caused by over-dominance and epistasis. In crosses involving high vs. low general combiners for yield components, mutual cancellation of heterosis components, especially dominance and its interaction, resulting in unfavorable SCA effects^17^. Crossing two parents with low general combiners produces high performance, attributable to complementary gene activity^27^. This study, SCA effects were shown to be significant for most yield characteristics. For base diameter, core diameter, fresh stem weight with leaves and pod, and fresh stem weight without leaves and pod, the best crossings suggested by SCA effects (Table 8) were P_2_ × P_3_, P_4_ × P_6_, and P_5_ × P_8_.

In contrast, the hybrids P_2_ × P_3_ and P_5_ × P_8_ were shown to be the best specific combiners for fiber yield, with high base diameter, core diameter, fresh stem weight with leaves and pod, and fresh stem weight without leaves and pod features. Furthermore, for stick yield and seed yield (plant number of pods), the hybrids P_1_ × P_4_ and P_7_ × were chosen as the best specific combiners. The hybrid P_2_ × P_5_ will be the best choice for 1st flowering and days to 50% flowering attributes in late maturity cultivars. In terms of another feature, the rest of the hybrids performed better. The strongest positive estimations of mid-parent and better parent heterosis for the qualities examined, indicating the accumulation of favorable genes inherited from their parental inbred lines. Among the hybrids produced, P_2_ (ML9) × P_3_ (ML36-10), P_5_ (ML36-25) × P_8_ (MLRing4P2), P_4_ (ML36-24) × P_6_ (ML36-27), P_1_ (ML5) × P_4_ (ML36-24), P_7_ (BJRI Kenaf4) × P_9_ [ML36-21(2)], P_2_ (ML9) × P_5_ (ML36-25), P_2_ (ML9) × P_9_ [ML36-21(2)] and P_1_ (ML5) × P_5_ (ML36-25) had higher yield and yield component qualities indicated by hybrids evaluated based on pooled data from the two environments.

The hybrids P_1_ × P_4_, P_4_ × P_6_ and P_7_ × P_9_ were chosen as the best specific combiners for dry stick weight and pods number per plant. Days to first flowering and days to 50% flowering for the hybrid P_2_ × P_5_ were also chosen as unique combiners. The best specific combiners for dry fiber weight and seeds number per pod were hybrid P_1_ × P_3_. For pods per plant and seeds per pod, the hybrids P_1_ × P_4_ and P_5_ × P_7_ were selected as good specific combiners. For another required attribute 1000 seed weight, the hybrids P_1_ × P_5_, P_3_ × P_8_, P_4_ × P_7_, and P_7_ × P_8_ were chosen as good specific combiners (smaller seed size). The data revealed that the GCA effects of parents were linked to the SCA impacts of their crossings, which had the greatest significant positive intensity. Complementing gene effects could explain the strong SCA effects of these crosses. Hybrid vigor can be induced by dominant, over-dominant, or epistatic gene action in any combination of parents, according to Moll and Stuber^28^. In this study, both additive and non-additive genetic components influenced morphological and yield-related traits, with non-additive gene activity dominating most of the characters.

Table 9 shows estimates of mid-parent and better parent heterosis for yield and yield component qualities indicated by hybrids evaluated based on pooled data from the two environments. Liu^25^ reported heterosis in yield characteristics based on the mid-parent and better parent. Heterosis estimates based on mid parental values were generally high, with stalk dry weight and bast percentage ranging from 10 to 55%^4^. The strongest positive estimations of mid-parent and better parent heterosis for the qualities examined were derived from P_4_ × P_6_ based on the combined data of the two environments (108.72% and 76.64%, respectively, for the pods number per plant). Hybrid P_2_ × P_3_ showed the greatest mid-parent and better parent heterosis for 4 of the 15 phenotypic parameters measured, including base diameter, core diameter, fresh stem weight with leaves and pods, and dry fiber weight, based on the combined data from the two environments.

In addition, hybrid P_5_ × P_8_ had the second highest mid-parent and better parent heterosis for base diameter, core diameter, middle diameter, fresh stem weight with leaves and pods, fresh stem weight without leaves and pods, and dry fiber weight, as well as P_4_ × P_6_ for base diameter, core diameter, fresh stem weight with leaves and pods, and pods number per plant. In terms of the features indicated, these hybrids outperformed their inbred parents. Hybrid P_7_ × P_9_ showed the better mid-parent and better parent heterosis for base diameter, core diameter, fresh stem weight with leaves and pods, and pods number per plant based on the combined data. Furthermore, the hybrid P_1_ × P_4_ was chosen as the best combiners for stick yield and pods number per plant, whereas hybrid P_4_ × P_9_ for stick yield and fiber yield showed the greatest mid-parent and better parent heterosis. In late maturity cultivars, the hybrid P_1_ × P_9_ will be the best choice for 1st flowering and days to 50% flowering features.

In contrast, hybrid P_2_ × P_9_ for Plant height, base diameter, core diameter, and fresh stem weight with leaves and pods showed low mid-parent and better parent heterosis estimates, followed by P_1_ × P_5_ for middle diameter, fresh stem weight without leaves and pods, and dry fiber weight, where the high values were unfavorable. Higher heterosis values compared to the better parent and the mid-parent suggested the absence of epistasis and the frequency of partial or total dominance of genes for fiber and seed yield. Crosses that produce superior transgressive segregants with higher fiber production could be found by looking at the percent F_1_ heterosis over the high parent.

The strongest positive estimations of mid-parent and better parent heterosis for the qualities examined, indicating the accumulation of favorable genes inherited from their parental inbred lines. Among the hybrids produced, P_2_ (ML9) × P_3_ (ML36-10), P_5_ (ML36-25) × P_8_ (MLRing4P2), P_4_ (ML36-24) × P_6_ (ML36-27), P_1_ (ML5) × P_4_ (ML36-24), P_7_ (BJRI Kenaf4) × P_9_ [ML36-21(2)], P_2_ (ML9) × P_5_ (ML36-25), P_2_ (ML9) × P_9_ [ML36-21(2)] and P_1_ (ML5) × P_5_ (ML36-25) had higher yield and yield component qualities indicated by hybrids evaluated based on pooled data from the two environments. Both additive and non-additive genetic components were essential in the control of many morphological and yield-related characters, with non-additive gene activities predominating for most of the characters.

## Conclusion

Additive and non-additive variations played a role in the genetic control of all variables in this study, including fiber yield and yield-related traits. New high-yielding kenaf hybrid types could be released via diallel selective mating or mass selection with simultaneous random mating. GCA effects were higher than SCA effects, except for top diameter, node number, and fresh stem weight with leaves and pod, as demonstrated by mean squares, showing that additive gene action predominates for these traits. Fiber yield and seed yield (number of pods per plant) are two important characteristics of kenaf production. Parent P_4_ (ML36-24) was the best general combiner for fiber yield (bast fiber) based on GCA performance, followed by P_1_ (ML5) and P_3_ (ML36-10). Conversely, P_4_ (ML36-24) was the best general combiner for stick yield (core fiber), followed by P_3_ (ML36-10). For the number of pods per plant (seed yield), P_7_ (BJRI Kenaf4) was shown to be the best general combiner, followed by P_6_ (ML36-27) and P_1_ (ML5). For days to 1st flowering and days to 50% flowering qualities, P_2_ (ML9) was chosen as an excellent general combiner. In a breeding programme, the hybrids P_2_ (ML9) × P_3_ (ML36-10), P_4_ (ML36-24) × P_6_ (ML36-27), and P_5_ (ML36-25) × P_8_ (MLRing4P2) will be the best for bast fiber and P_1_ (ML5) × P_4_ (ML36-24), P_5_ (ML36-25) × P_8_ (MLRing4P2), and P_7_ (BJRI Kenaf4) × P_9_ [ML36-21(2)] will be the best for core fiber if fiber yield is the most significant selection factor. If seed yield is important in the breeding programme, the hybrids P_1_ (ML5) × P_4_ (ML36-24), P_4_ (ML36-24) × P_6_ (ML36-27), and P_7_ (BJRI Kenaf4) × P_9_ [ML36-21(2)] will be the best choices for number of pods per plant, and the hybrid P_2_ (ML9) × P_5_ (ML36-25) will be the best choice for days to first flowering and days to 50% flowering traits for late maturity cultivars. Crossings of P_1_ (ML5) × P_4_ (ML36-24), P_1_ (ML5) × P_9_ [ML36-21(2)], P_2_ (ML9) × P_3_ (ML36-10), P_2_ (ML9) × P_5_ (ML36-25), P_4_ (ML36-24) × P_6_ (ML36-27), P_4_ (ML36-24) × P_7_ (BJRI Kenaf4), P_4_ (ML36-24) × P_9_ [ML36-21(2)], P_5_ (ML36-25) × P_8_ (MLRing4P2), and P_7_ (BJRI Kenaf4) × P_9_ [ML36-21(2)] showed promising heterotic responses and could benefit future breeding programs.

## Supplementary Information

Supplementary Figure 1.
