# Supplementary material for: Combining ability and gene action for yield improvement in kenaf (Hibiscus cannabinus L.) under tropical conditions through diallel mating design
Source: Sci Rep. 2022 Jun 10;12:9646. doi: 10.1038/s41598-022-13529-y (PMC9187750; doi:10.1038/s41598-022-13529-y)
Supplement: Supplementary file 1 — Supplementary Figure 1. [file 41598_2022_13529_MOESM1_ESM.docx]

| ♀ | ♂ | F_1_ | ♀ | ♂ | F_1_ |
| --- | --- | --- | --- | --- | --- |
| 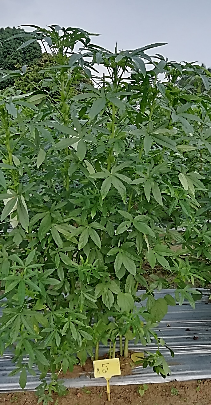 | 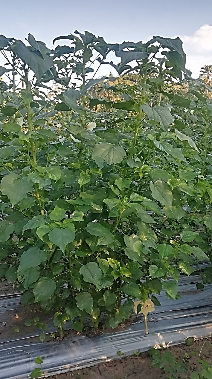 | 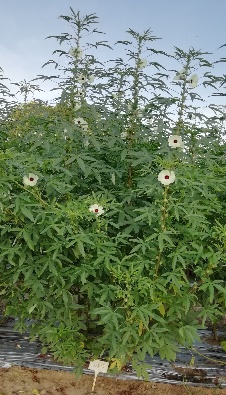 | 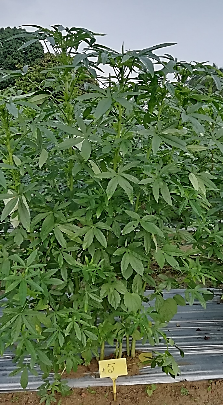 | 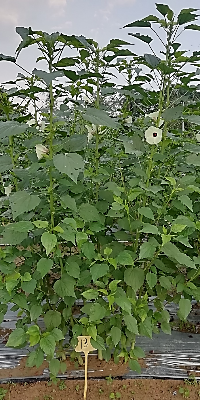 | 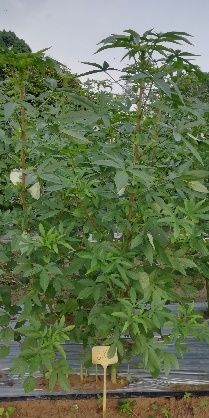 |
| P_1_ | P_2_ | P_1_ × P_2_ | P_1_ | P_3_ | P_1_ × P_3_ |
| 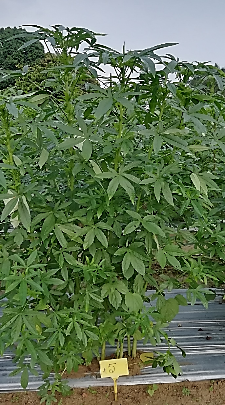 | 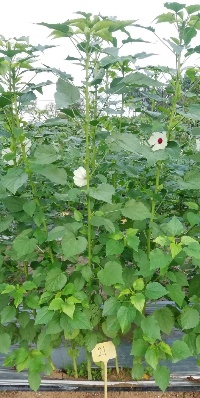 | 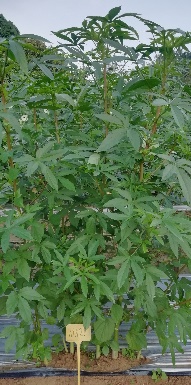 | 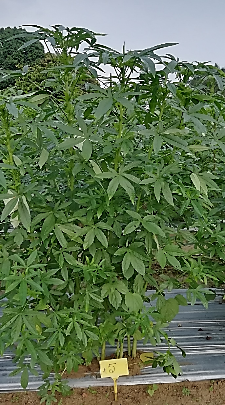 | 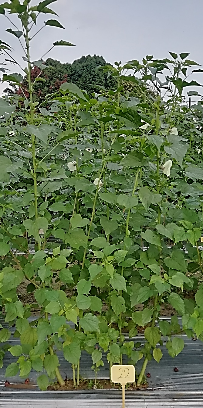 | 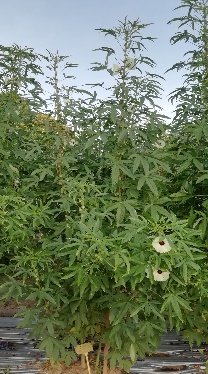 |
| P_1_ | P_4_ | P_1_ × P_4_ | P_1_ | P_5_ | P_1_ × P_5_ |
| 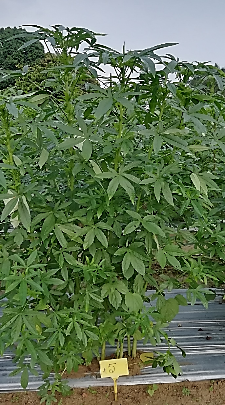 | 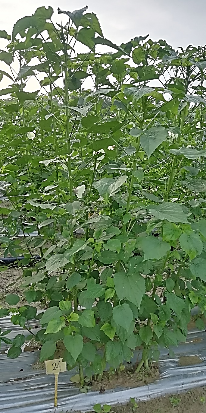 | 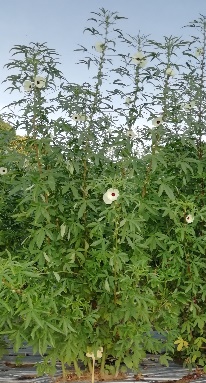 | 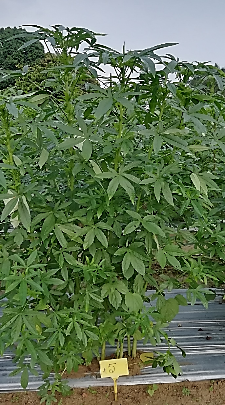 | 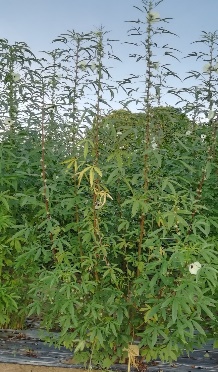 | 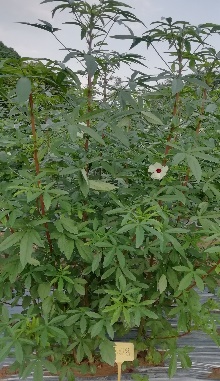 |
| P_1_ | P_6_ | P_1_ × P_6_ | P_1_ | P_7_ | P_1_ × P_7_ |
| 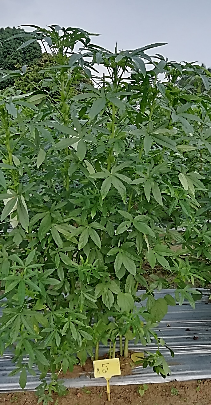 | 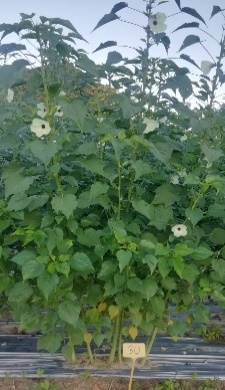 | 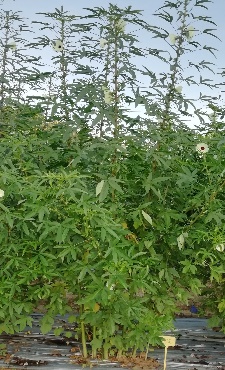 | 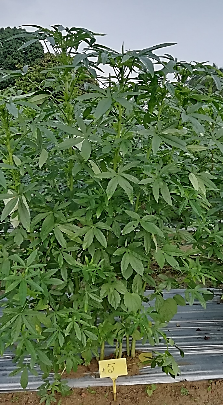 | 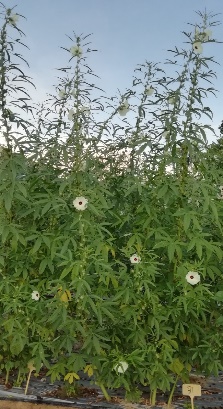 | 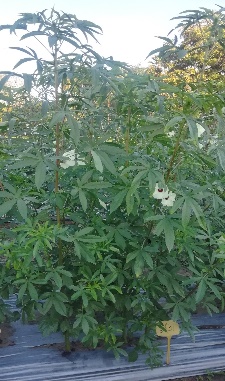 |
| P_1_ | P_8_ | P_1_ × P_8_ | P_1_ | P_9_ | P_1_ × P_9_ |
| 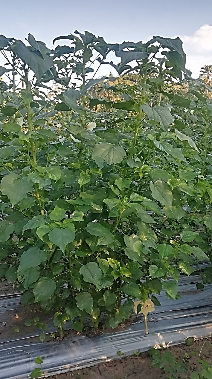 | 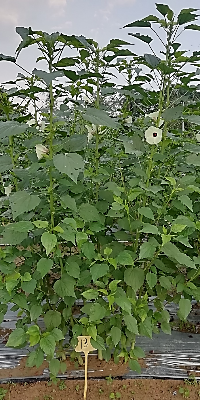 | 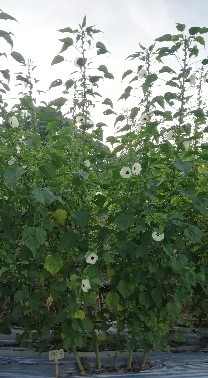 | 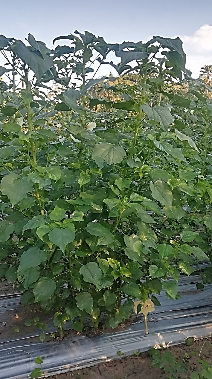 | 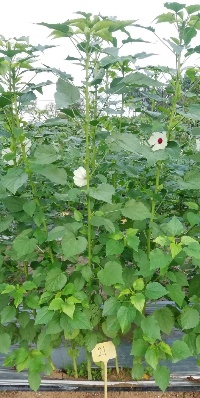 | 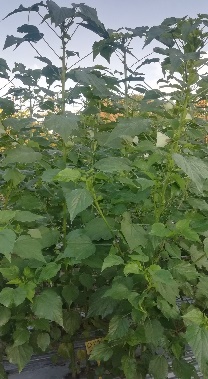 |
| P_2_ | P_3_ | P_2_ × P_3_ | P_2_ | P_4_ | P_2_ × P_4_ |

**Supplementary figure 1.** Comparison of photographs showing stem pigmentation and leaf shape of parents and F_1_ population

| ♀ | ♂ | F_1_ | ♀ | ♂ | F_1_ |
| --- | --- | --- | --- | --- | --- |
| 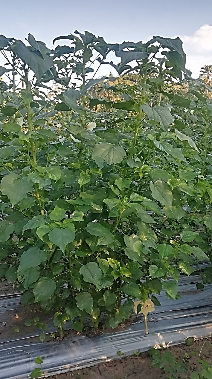 | 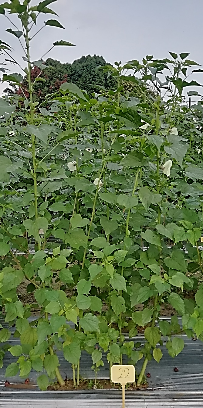 | 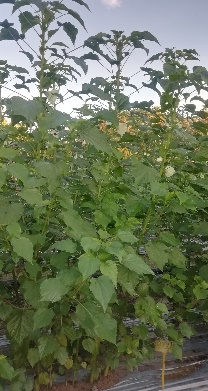 | 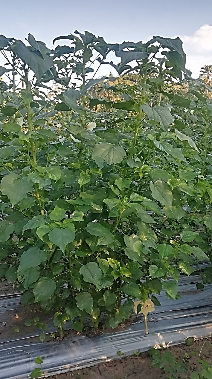 | 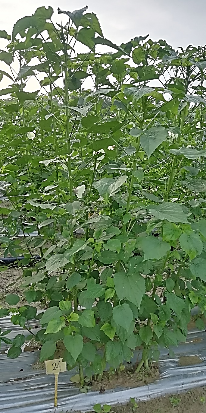 | 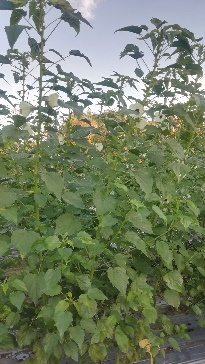 |
| P_2_ | P_5_ | P_2_ × P_5_ | P_2_ | P_6_ | P_2_ × P_6_ |
| 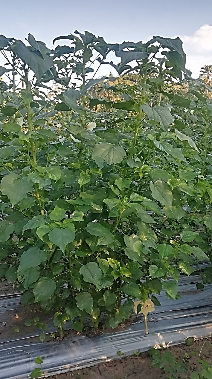 | 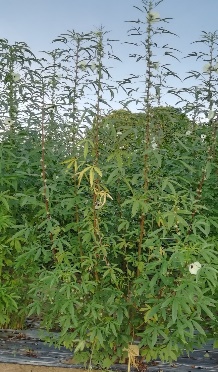 | 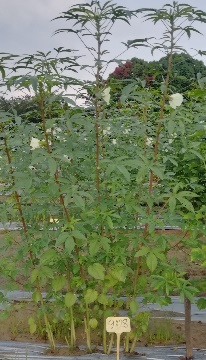 | 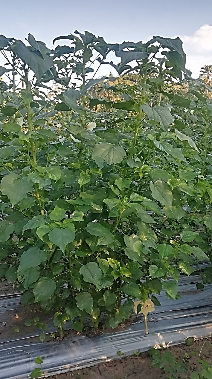 | 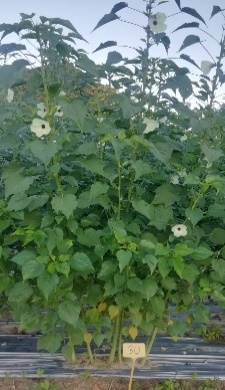 | 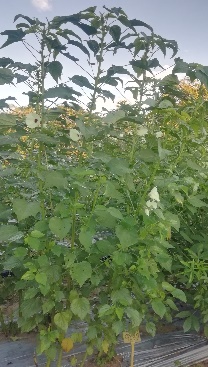 |
| P_2_ | P_7_ | P_2_ × P_7_ | P_2_ | P_8_ | P_2_ × P_8_ |
| 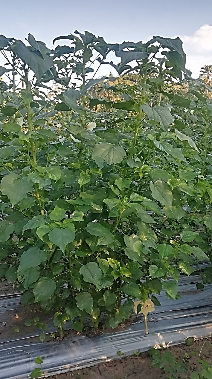 | 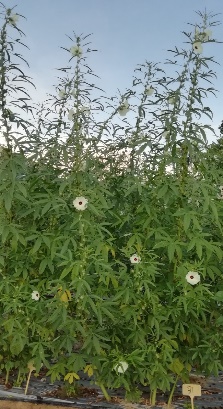 | 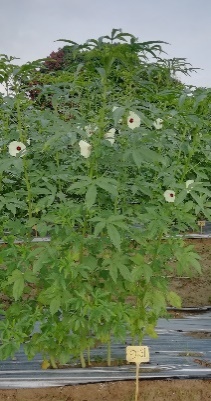 | 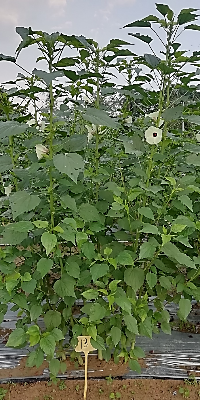 | 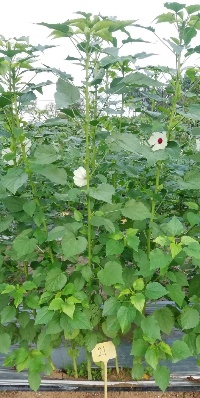 | 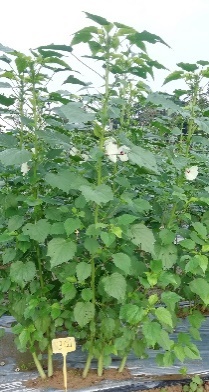 |
| P_2_ | P_9_ | P_2_ × P_9_ | P_3_ | P_4_ | P_3_ × P_4_ |
| 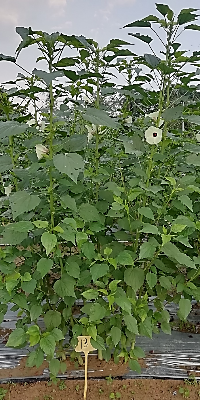 | 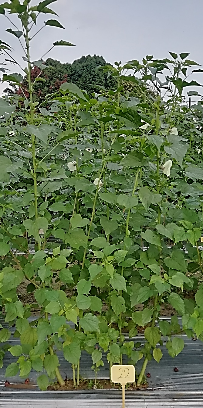 | 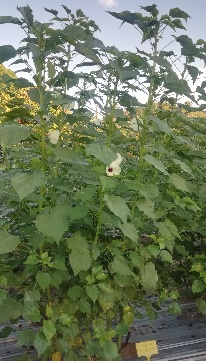 | 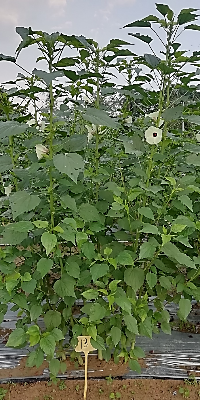 | 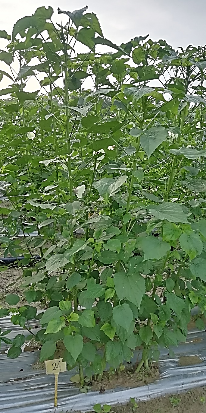 | 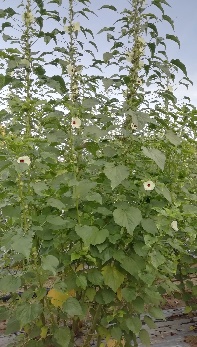 |
| P_3_ | P_5_ | P_3_ × P_5_ | P_3_ | P_6_ | P_3_ × P_6_ |
| 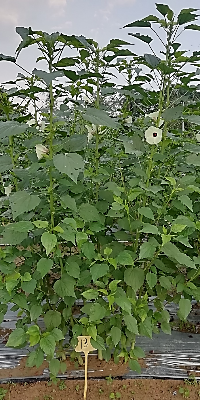 | 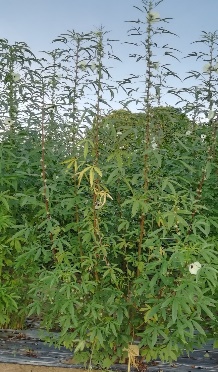 | 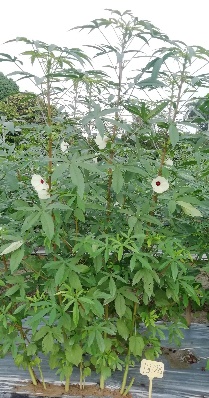 | 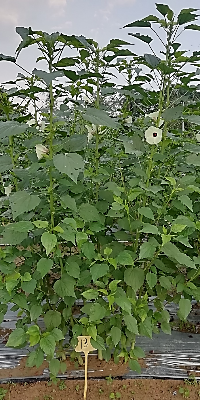 | 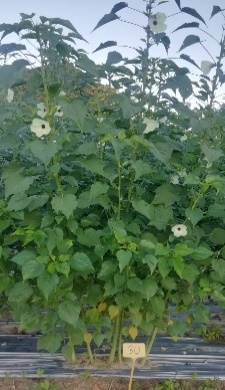 | 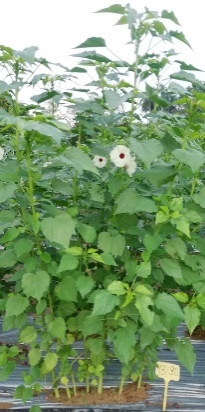 |
| P_3_ | P_7_ | P_3_ × P_7_ | P_3_ | P_8_ | P_3_ × P_8_ |

Cont’d. (Supplementary figure 1)

| ♀ | ♂ | F_1_ | ♀ | ♂ | F_1_ |
| --- | --- | --- | --- | --- | --- |
| 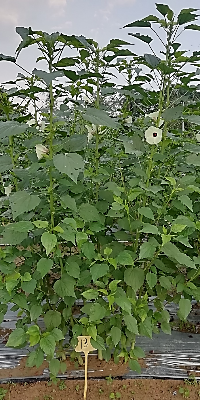 | 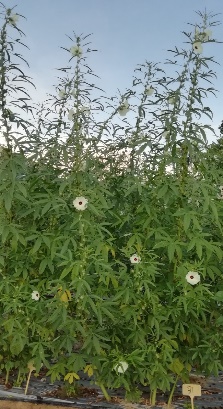 | 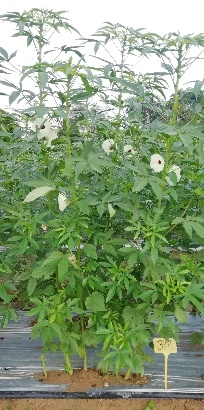 | 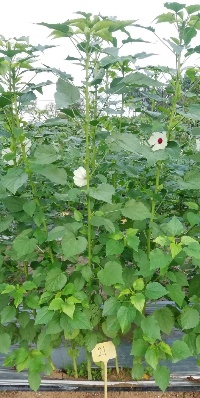 | 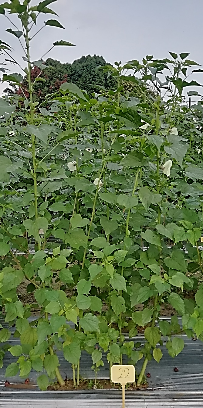 | 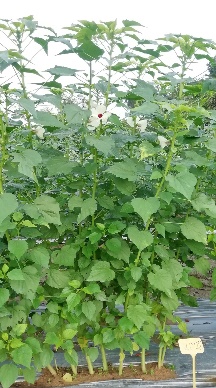 |
| P_3_ | P_9_ | P_3_ × P_9_ | P_4_ | P_5_ | P_4_ × P_5_ |
| 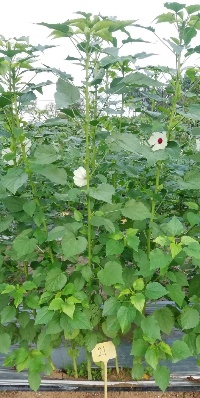 | 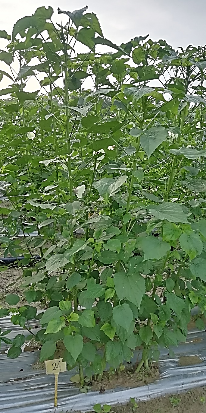 | 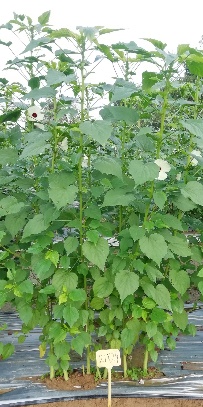 | 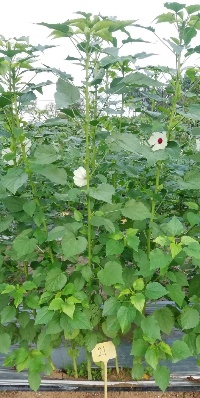 | 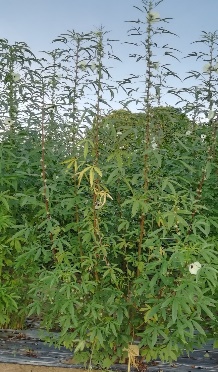 | 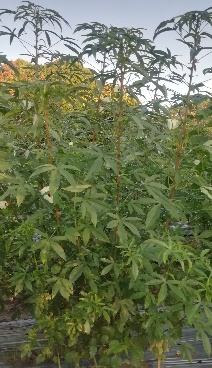 |
| P_4_ | P_6_ | P_4_ × P_6_ | P_4_ | P_7_ | P_4_ × P_7_ |
| 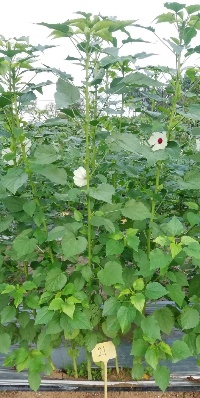 | 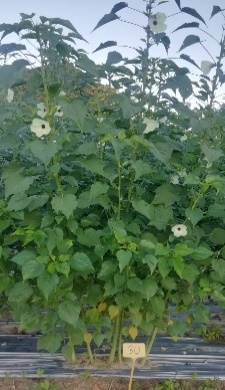 | 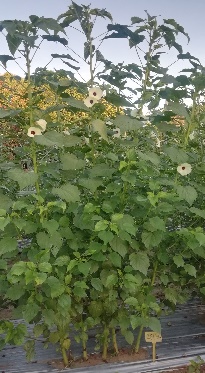 | 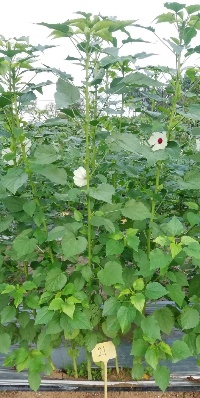 | 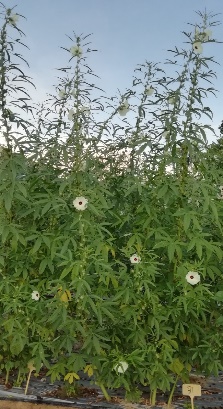 | 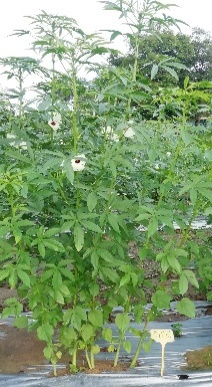 |
| P_4_ | P_8_ | P_4_ × P_8_ | P_4_ | P_9_ | P_4_ × P_9_ |
| 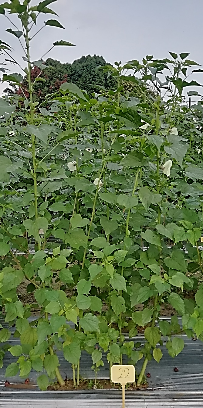 | 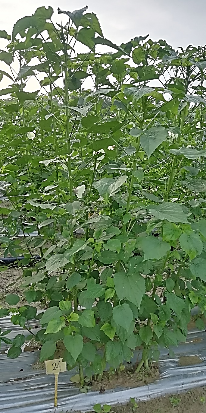 | 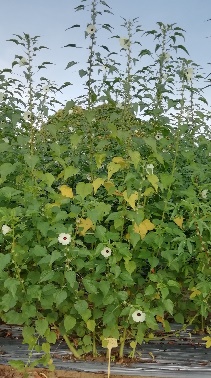 | 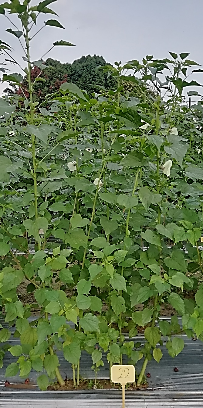 | 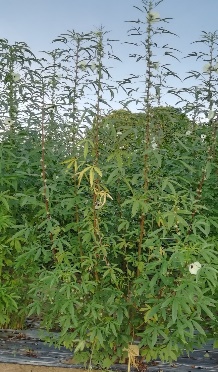 | 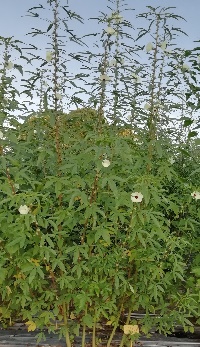 |
| P_5_ | P_6_ | P_5_ × P_6_ | P_5_ | P_7_ | P_5_ × P_7_ |
| 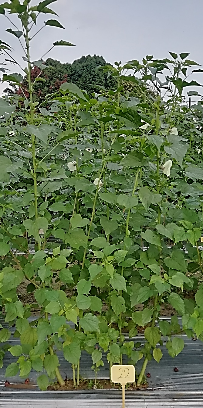 | 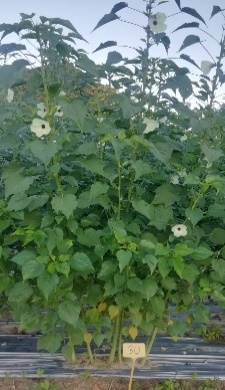 | 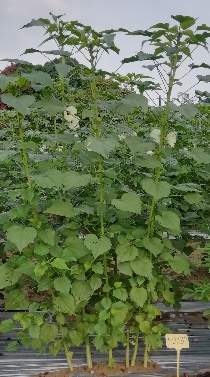 | 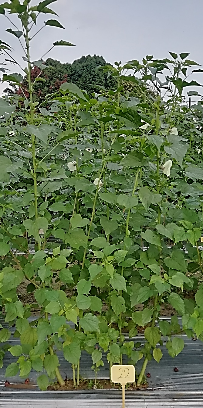 | 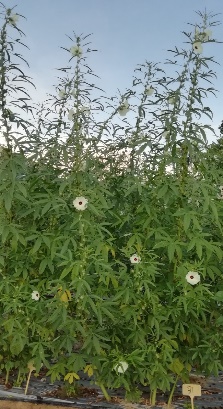 | 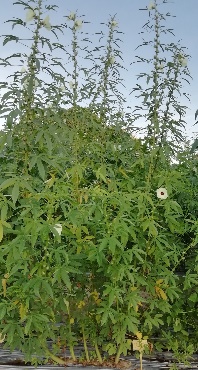 |
| P_5_ | P_8_ | P_5_ × P_8_ | P_5_ | P_9_ | P_5_ × P_9_ |

Cont’d. (Supplementary figure 1)

| ♀ | ♂ | F_1_ | ♀ | ♂ | F_1_ |
| --- | --- | --- | --- | --- | --- |
| 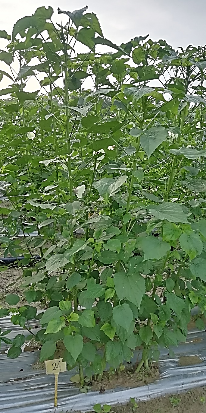 | 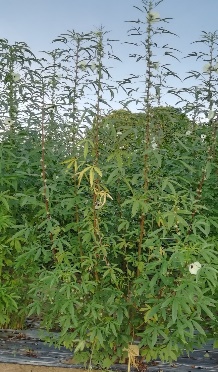 | 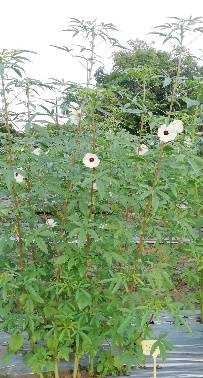 | 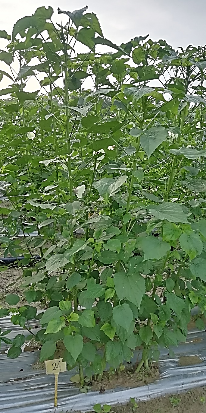 | 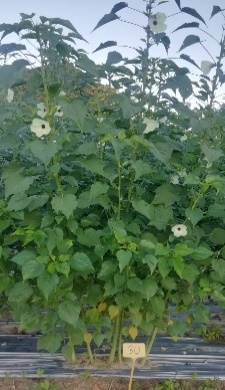 | 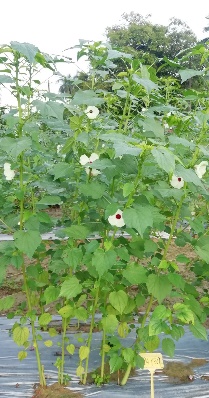 |
| P_6_ | P_7_ | P_6_ × P_7_ | P_6_ | P_8_ | P_6_ × P_8_ |
| 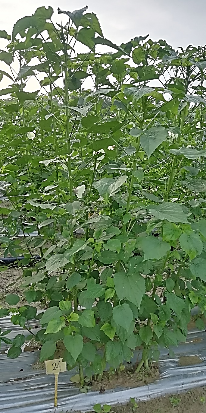 | 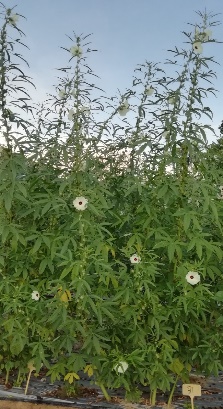 | 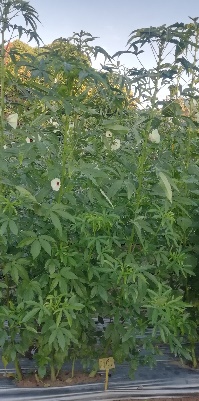 | 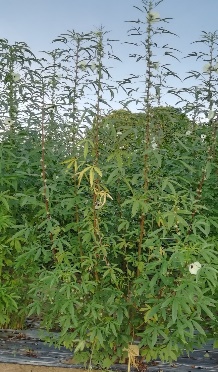 |  |  |
| P_6_ | P_9_ | P_6_ × P_9_ | P_7_ | P_8_ | P_7_ × P_8_ |
|  |  |  |  |  |  |
| P_7_ | P_9_ | P_7_ × P_9_ | P_8_ | P_9_ | P_8_ × P_9_ |

Cont’d. (Supplementary figure 1)
